# MYC-dependent upregulation of the de novo serine and glycine synthesis pathway is a targetable metabolic vulnerability in group 3 medulloblastoma

**DOI:** 10.1093/neuonc/noae179

**Published:** 2024-10-08

**Authors:** Magretta Adiamah, Bethany Poole, Janet C Lindsey, Sarah Kohe, Alaide Morcavallo, Florence Burté, Rebecca M Hill, Helen Blair, Dean Thompson, Mankaran Singh, Shanel Swartz, Stephen Crosier, Tong Zhang, Oliver D K Maddocks, Andrew Peet, Louis Chesler, Ian Hickson, Ross J Maxwell, Steven C Clifford

**Affiliations:** Wolfson Childhood Cancer Research Centre, Newcastle University Centre for Cancer, Newcastle Upon Tyne, UK; Wolfson Childhood Cancer Research Centre, Newcastle University Centre for Cancer, Newcastle Upon Tyne, UK; Wolfson Childhood Cancer Research Centre, Newcastle University Centre for Cancer, Newcastle Upon Tyne, UK; Institute of Cancer and Genomic Sciences, University of Birmingham, Birmingham, UK; Division of Clinical Studies, Institute of Cancer Research (ICR), London and Royal Marsden NHS Trust, Sutton, UK; Wolfson Childhood Cancer Research Centre, Newcastle University Centre for Cancer, Newcastle Upon Tyne, UK; Wolfson Childhood Cancer Research Centre, Newcastle University Centre for Cancer, Newcastle Upon Tyne, UK; Wolfson Childhood Cancer Research Centre, Newcastle University Centre for Cancer, Newcastle Upon Tyne, UK; Wolfson Childhood Cancer Research Centre, Newcastle University Centre for Cancer, Newcastle Upon Tyne, UK; Wolfson Childhood Cancer Research Centre, Newcastle University Centre for Cancer, Newcastle Upon Tyne, UK; Wolfson Childhood Cancer Research Centre, Newcastle University Centre for Cancer, Newcastle Upon Tyne, UK; Wolfson Childhood Cancer Research Centre, Newcastle University Centre for Cancer, Newcastle Upon Tyne, UK; Institute of Cancer Sciences, University of Glasgow, Glasgow, UK; Institute of Cancer Sciences, University of Glasgow, Glasgow, UK; Institute of Cancer and Genomic Sciences, University of Birmingham, Birmingham, UK; Division of Clinical Studies, Institute of Cancer Research (ICR), London and Royal Marsden NHS Trust, Sutton, UK; Wolfson Childhood Cancer Research Centre, Newcastle University Centre for Cancer, Newcastle Upon Tyne, UK; Wolfson Childhood Cancer Research Centre, Newcastle University Centre for Cancer, Newcastle Upon Tyne, UK; Wolfson Childhood Cancer Research Centre, Newcastle University Centre for Cancer, Newcastle Upon Tyne, UK

**Keywords:** PHGDH, MYC, medulloblastoma, metabolism, serine

## Abstract

**Background:**

Group 3 medulloblastoma (MB_GRP3_) represents around 25% of medulloblastomas and is strongly associated with *c-MYC* (*MYC*) amplification, which confers significantly worse patient survival. Although elevated *MYC* expression is a significant molecular feature in MB_GRP3_, direct targeting of MYC remains elusive, and alternative strategies are needed. The metabolic landscape of MYC-driven MB_GRP3_ is largely unexplored and may offer novel opportunities for therapies.

**Methods:**

To study MYC-induced metabolic alterations in MB_GRP3_, we depleted MYC in isogenic cell-based model systems, followed by ^1^H high-resolution magic-angle spectroscopy (HRMAS) and stable isotope-resolved metabolomics, to assess changes in intracellular metabolites and pathway dynamics.

**Results:**

Steady-state metabolic profiling revealed consistent MYC-dependent alterations in metabolites involved in one-carbon metabolism such as glycine. ^13^C-glucose tracing further revealed a reduction in glucose-derived serine and glycine (de novo synthesis) following MYC knockdown, which coincided with lower expression and activity of phosphoglycerate dehydrogenase (PHGDH), the rate-limiting enzyme in this pathway. Furthermore, MYC-overexpressing MB_GRP3_ cells were more vulnerable to pharmacological inhibition of PHGDH compared to those with low expression. Using in vivo tumor-bearing genetically engineered and xenograft mouse models, pharmacological inhibition of PHGDH increased survival, implicating the de novo serine/glycine synthesis pathway as a pro-survival mechanism sustaining tumor progression. Critically, in primary human medulloblastomas, increased PHGDH expression correlated strongly with both *MYC* amplification and poorer clinical outcomes.

**Conclusions:**

Our findings support a MYC-induced dependency on the serine/glycine pathway in MB_GRP3_ that represents a novel therapeutic treatment strategy for this poor prognosis disease group.

## Graphical Abstract

**Graphical Abstract:**
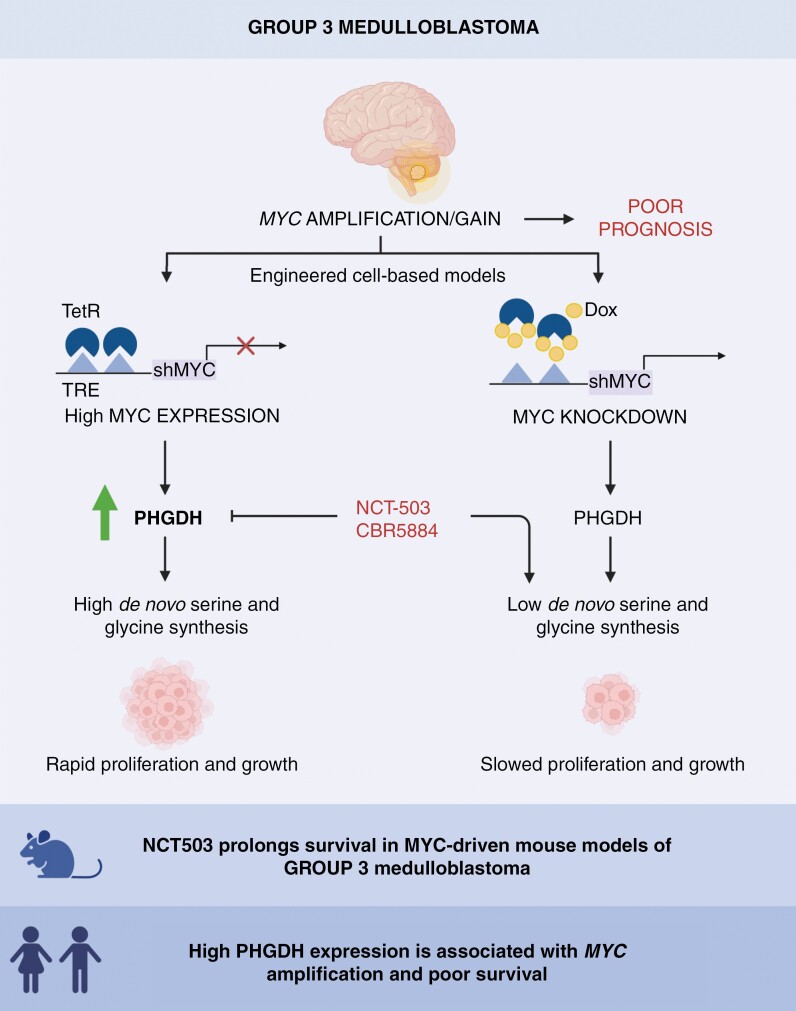

**Key Points**

1. Serine synthesis is upregulated in MYC-driven MB_GRP3_ models via PHGDH.
2. MYC-driven PHGDH overexpression enhances sensitivity to PHDGH inhibition in vitro and in vivo.
3. In primary tumors, PHGDH expression is associated with MYC-MB_GRP3_ and worse outcomes.

**Importance of Study**

*MYC* amplification is the most significant poor prognosis biological feature in Group 3 medulloblastoma (MB_GRP3_). However, there is a lag in the development of targeted therapies and these tumors remain refractory to current multimodal therapies. While direct MYC targeting is challenging, MYC regulation of diverse cellular processes creates dependencies which are more amenable to therapeutic targeting. We performed metabolic profiling and further phenotypic characterization in diverse MYC-dependent models and identified MYC-induced upregulation of the de novo serine/glycine pathway with increased sensitivity to PHDGH inhibitors. Additionally, we observed elevated PHDGH expression linked with *MYC* amplification in patients and show that increased PHDGH overexpression is an independent prognostic factor linked with worse survival in medulloblastoma and MB_GRP3_ specifically. The clinical relevance of our findings provides a strong basis for further exploration of PHGDH inhibition and other alternatives in limiting serine/glycine pathway activity in MYC-driven MB_GRP3_.

Medulloblastoma is one of the most frequently diagnosed malignant brain tumors in the pediatric population and contributes to a significant proportion of childhood cancer deaths.^1^ Extensive molecular classification of medulloblastoma has defined four major consensus molecular groups based on distinct genomic features, disease demographics, and survival outcomes.^2–4^ The Wingless (MB_WNT_) and Sonic Hedgehog (MB_SHH_) subgroups display abnormalities in their respective developmental pathways. Group 3 (MB_GRP3_) and Group 4 (MB_GRP4_) however are not defined by a distinct pathway and display significant intertumoral heterogeneity.^5^ MB_GRP3_ tumors may be highly anaplastic with an increased propensity for metastatic spread and, overall, exhibit poorer survival outcomes.^6^ The most notable feature of MB_GRP3_ is the high expression of *c-MYC* (*MYC*), with bona fide genomic amplifications of *MYC* observed in around 20% of MB_GRP3._*MYC*-amplification drives aggressive disease in several MB_GRP3_ mouse models.^7,8^ MB_GRP3_ patients subsequently undergo intense multimodal treatments which often fail to combat disease but are associated with significant neurocognitive deficits and side effects.^9^ The considerable advancements in medulloblastoma molecular pathology and risk stratification have translated to subgroup-specific therapies for MB_WNT_^10^ and MB_SHH_ subgroups,^11^ but the development of targeted treatments for MB_GRP3_ is limited. Understanding the role of MYC and its dependencies in MB_GRP3_ tumors, particularly in cellular processes such as metabolism, is largely unexplored and may yield new therapeutic opportunities.

The MYC family of transcription factors (MYC, MYCN, and MYCL) oversee large transcriptional networks regulating cellular processes such as ribosome biogenesis, translation, metabolism, and cell cycle progression, to support proliferative and differentiation programs, amongst others.^12,13^ In MYC-driven tumors, this broad transcriptional network is co-opted to facilitate and maintain abnormal cancer growth,^14^ and evidence of these MYC-associated hyperproliferative programs has been observed in large-scale tumor proteomic profiling studies.^15,16^ Although frequently altered in cancer, the inability to directly target MYC has focused efforts on its dependencies.^17^ Previous approaches have targeted features such as MYC expression and stability, cell cycle progression, and replication stress, as routes for slowing tumor proliferation/growth using mono/combination therapies.^14,18^

Metabolic enzymes are among the transcriptional targets of MYC and represent a further potential approach to indirectly target MYC in MB_GRP3._ In first studies, a high-throughput drug screen showed *MYC*-amplified MB_GRP3_ cells were more sensitive to the combination of gemcitabine and permetrexed (inhibitor of folate metabolism) compared to MB_SHH_ cells.^19^ Additionally, studies of a recently developed MYC-driven MB_GRP3_ mouse model revealed an association with LDHA overexpression. Crucially, LDHA inhibition slowed the growth of MYC-driven tumors but had minimal impact on normal cerebellar cells or MB_SHH,_ indicating distinct metabolic programs in MB_GRP3_.^20^ However, the wider metabolic landscape of medulloblastoma remains poorly understood and metabolic profiling studies to support rational target identification are, to date, limited.

Previously, ^1^H high-resolution magic-angle spectroscopy (HRMAS) has been shown to distinguish embryonal tumor types using metabolite profiles alone^21^ and more recently, has been used to distinguish molecular subgroups of medulloblastoma.^22^ Indeed, these altered metabolic signatures also suggested crucial metabolic pathways fueling these tumors. In this study, we therefore employed HRMAS to determine metabolic signatures intimately linked with *MYC* amplification or gain in MB_GRP3_. Using novel *MYC*-regulable cell-based models, we sought to uncover MYC-dependent metabolic alterations and reveal novel targetable dependencies. We show MYC-dependent MB_GRP3_ cells are dependent on the de novo serine and glycine pathway to fuel their proliferative capacity and are vulnerable to its genetic and pharmacological perturbation in in vitro and in vivo preclinical models. Furthermore, expression of phosphoglycerate dehydrogenase (PHGDH), the committing step of glucose-derived 3-phosphoglycerate towards de novo serine and glycine synthesis, is correlated with *MYC* amplification and poor survival in primary human MB_GRP3_ tumors. Together, our findings reveal enhanced de novo serine biosynthesis driven by MYC as a novel metabolic adaptation in tumor development, with significant potential as a clinically relevant and actionable therapeutic vulnerability for MYC-driven MB_GRP3_.

## Materials and Methods

### Cell Culture

**Figure 1.**
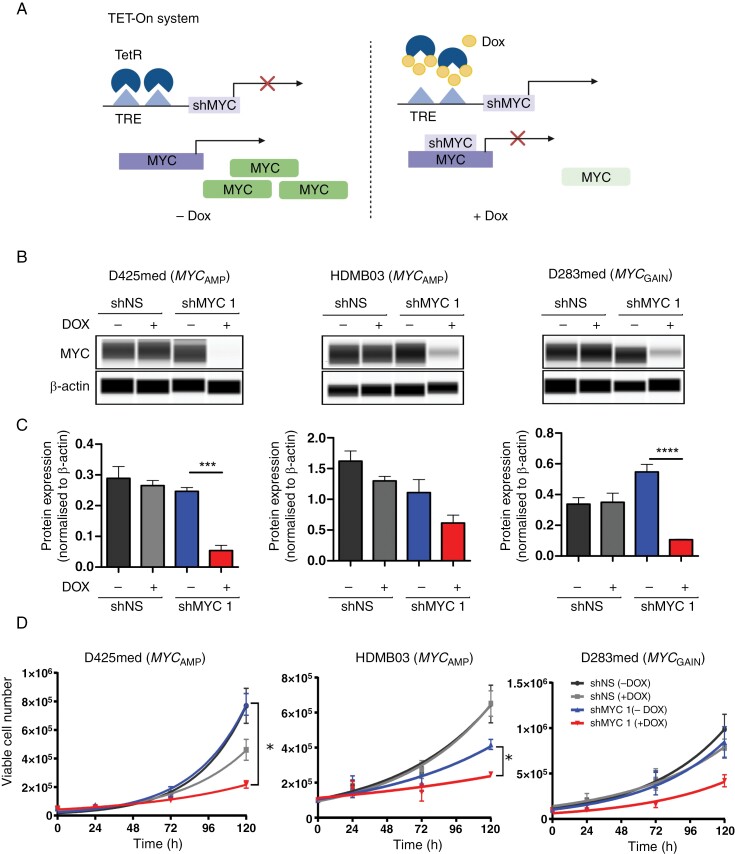
Downregulation of MYC expression in MYC-driven MB_GRP3_ models shows retained MYC addiction. (A) Schematic diagram demonstrating doxycycline-inducible knockdown of MYC expression. TetR, Tetracycline; TRE, Trans-regulatory elements (created using Biorender.com). (B) Immunoblot analysis and (C) quantification of MYC protein expression in D425med, HDMB03, and D283med medulloblastoma cells expressing shRNAs targeting non-silencing (shNS) and MYC (shMYC1) upon addition of 1 µg/mL doxycycline (Dox) which induces MYC knockdown at 72 hours. β-actin was used as a loading control. (D) Effect of MYC knockdown on cell proliferation was assessed using trypan blue dye exclusion viable cell counting over 120 hours. Growth curves depict D425med, HDMB03, D283med shNS, and shMYC1 bearings cells ± Dox. Values are expressed as mean ± SEM of 3 biological replicates. *P < .05, **P < .01, ***P < .001,**** *P* < .0001.

*MYC*-amplified MB_GRP3s_ cell lines D425med (a gift from Dr Bigner, Duke University), D283med (Acquired from ATCC), and HDMB03 (a gift from Dr Deubzer, German Cancer Research Center (DKFZ)) were transduced with vectors containing tetracycline (TET) inducible shRNA (Tet-pLKO-puro was a gift from Dmitri Wiederschain [Addgene plasmid # 21915]); 2 independent doxycycline-inducible shRNA constructs targeting MYC for knockdown (shMYC1,2) and a non-silencing shRNA construct which served as control (shRNA targeting sequences are outlined in Supplementary Table 1). Isogenic cell lines were routinely cultured in Dulbecco’s modified eagle medium (DMEM; Sigma Aldrich, #D6171) or Roswell Park Memorial Institute medium 1640 medium (RPMI; Sigma Aldrich, #R5886) and supplemented with 10% tetracycline free FBS (Takara Bio, #63110 6) 1% l-glutamine (#G7513) and 1 µg/mL puromycin (ThermoFisher, # A1113803). The addition of doxycycline to the growth medium triggered shRNA expression causing MYC knockdown with shMYC1 and 2 (Figure 1A). Cell line identity was previously karyotyped and confirmed.^23^

### Growth Curves

Cells were plated at a density of 1 × 10^5^ cells/well into 6-well plates and viable cell number counting was performed using trypan blue dye exclusion.

### Immunoblotting

Protein extracts from cells were harvested and immunoblotted using standard methods. Protein lysates were loaded into WES (Protein Simple) plates at 0.8 µg per well. Assay plates were prepared according to the manufacturer’s instructions (Bio-Techne). Antibodies and dilutions are outlined in Supplementary Methods.

### HRMAS Metabolite Profiling

Cells were scraped, washed twice in ice-cold PBS, and centrifuged at 200 *g* for 5 minutes at 4 °C. Cell pellets were flash-frozen in dry ice. Approximately 40 µL of thawed cell pellet was placed into a zirconium rotor and 5 µL of D_2_0 with 10 mM TMSP (Cambridge Biosciences) as an internal standard. ^1^H spectra were acquired in a 500 MHz Bruker AVANCE spectrometer. Detailed further in Supplementary Methods.

### RNA Sequencing

For all isogenic shMYC and shNS cell lines, RNA was extracted and sequenced as previously described.^24^

### PHGDH Activity Assay

PHGDH activity was assessed using the Phosphoglycerate Dehydrogenase (PHGDH) Activity Assay Kit (Abcam; ab273328) according to the manufacturer’s instructions.

### ^13^C-Glucose Labeling

D425med shMYC1 cells with pretreatment of 1 µg/mL of doxycycline for 48 hours were cultured in glucose-free DMEM supplemented with dialyzed 10% FBS (ThermoFisher, # A3382001) and 10 mM D-glucose-^13^C (Sigma-Aldrich, UK # 389374) with or without doxycycline for a further 6 or 24 hours. Cells were directly scraped into the extraction mixture and stored at −80 °C. Detailed further in supplementary methods.

### Cytotoxicity Assays

Medulloblastoma cell lines were cultured under their standard culture conditions and treated with NCT-503 (Sigma #SML1659) or CBR5884 for 72 or 96 hours alongside appropriate controls. Cell viability was determined using CellTiter Glo 2.0 (G9241 Promega).

### Colony Formation Assays

Parental medulloblastoma cells treated with NCT-503 were seeded at 5 × 10^2^ cells/well in 6-well culture plates. Cells were incubated for up to 7 days to allow colony formation. Colonies were fixed using methanol on ice and stained with 0.01% (w/v) crystal violet. Where appropriate, colonies were counted using a stereomicroscope and digital images were acquired using a camera device.

### Cell Cycle Analysis

Cells were fixed in 1 mL of cold 70% (v/v) ethanol at 4 °C for at least 30 minutes. Cells were washed twice in PBS and then re-suspended in 0.5 mL of PBS. Cells were treated with 50 µL of Ribonuclease A (RNAase; Thermo Fisher) and stained with 50 µg/mL of propidium iodide. PI fluorescence (605 nm) was measured on an Attune NxT cytometer (Thermo Fisher) and analyzed using FlowJo Software (version 10.6, BD Life Sciences).

### In Vivo Studies

All experimental protocols were approved by the Animal Welfare and Ethical Review Body at Newcastle University (project license number P74687DB5) and The Institute of Cancer Research (P91E52C32) in compliance with specified guidelines outlined by the UK Home Office Animals (Scientific Procedures) Act 1986. *Subcutaneous xenografts:* 5 × 10^5^ D425med shMYC1 cells mixed with matrigel (Corning #354234) at 1:1 ratio were implanted subcutaneously in NOD-scid gamma mice. Four days post-implantation, NCT-503 (40 mg/kg) or vehicle control was delivered via daily intraperitoneal injections until the humane endpoint (tumors reaching 15 mm). NCT-503 was prepared in a vehicle of 5% ethanol, 35% PEG-300 (Sigma), and 60% of a 30% (w/v) hydroxypropyl-β-cyclodextrin (ThermoFisher UK, #297565000). Tumor growth was measured using calipers on a weekly basis. Tumor volume was calculated using the following formula: $\left(length\;\times width^{2}\right)/2$. *Genetically engineered mouse model:* Transgenic GTML *Trp53*^KI/KI25^ mice were injected with D-luciferin (75 mg/kg) prior to bioluminescence imaging. Animals with tumors reaching 3 × 10^9^ photons/secs (20–30 days of life) were randomized into control and NCT-503 treatment groups with daily dosing for up to 24 days. Animals were routinely monitored and sacrificed upon meeting clinical scoring criteria associated with high tumor burden. Mice had access to food and water ad libitum.

### Gene Expression

Microarray data for medulloblastoma patient samples was obtained from the R2 genomics platform (https://hgserver1.amc.nl/cgi-bin/r2/main.cgi) and was used to determine differentially expressed genes according to the KEGG-defined genes of the “serine_glycine_threonine” pathway. The cohort included 144 MB_GRP3_ samples including relevant clinical data and prognostic factors such as subgroup classification.^2^ We identified 17 differentially altered genes in the serine_glycine_threonine pathway which was later defined as the “SGP gene signature” for further analysis.

### Analysis of Newcastle MB_GRP3_ Cohort

Previously generated RNA-seq data from primary MB_GRP3_ patient samples (*n* = 36), which included both *MYC*-amplified (*n* = 9) and non-amplified (*n* = 27) MB_GRP3_ was used as an independent cohort to assess gene expression changes in the SGP pathway.^24^ Hierarchical clustering and pathway analysis were performed using R studio statistical packages GSEA, fGSEA, and PHeatmap (R version 4.0.2).

### Immunohistochemical Analysis

Core tissue biopsies from individual patient formalin fixed paraffin embedded medulloblastoma tumors were arrayed in duplicate using an automated tissue processor (Leica TP1020 semi-enclosed benchtop tissue processor) to form a tissue microarray (TMA). Additionally, samples from normal cerebellum, spleen, placenta, and tumor samples from colon and prostate adenocarcinomas were added to each TMA to act as controls for immunohistochemical staining. The overall cohort analysis was based on diagnostic medulloblastoma samples (*n* = 183; Supplementary Figure 7b). Paraffin-embedded TMA slide sections were stained according to standard protocols using heat-induced antigen retrieval. Slides were incubated with PHGDH antibody (1:100; Proteintech, UK #14719-1-AP) and antigen detection using Menapath X-Cell plus HRP-polymer kit (A.Menarini diagnostics, #MP-860).

### Statistical Analysis

All experiments were performed using a minimum of 3 biological replicates, and statistical analysis derived from these replicates. For in vivo experiments, animals were randomly assigned to treatment groups. Statistical analysis was carried out using student *t*-test, ANOVA, and linear regression using Graphpad Prism (Version 9.4). Biological/technical replicates along with specific statistical analysis are indicated in figure legends. Statistical significance is denoted by asterisks (* *P* < .05,** *P* < .01, *** *P* < .001, **** *P* < .0001). For clinical data, survival analysis using Kaplan–Meier curves and log-rank test, and Cox-proportional hazard regression, were performed using R (version 4.0.2) packages “survival” and “survminer.”

Additional information can be found in Supplementary Materials and Methods.

## Results

### MB_GRP3_ Models Display MYC Addiction

We first developed 3 independent cell-based models of MYC-dependent MB_GRP3_. Three MYC-overexpressing MB_GRP3_ cell lines—D425med,^26^ HDMB03^27^ (both *MYC* amplified), and D283med (*MYC* gain)^28^—were transduced with lentivirus containing a Tet-on vector with shRNA targeting sequences under the control of a tetracycline-responsive (ie, doxycycline [Dox]-inducible) promoter (Figure 1A).^29^ Two independent anti-*MYC* shRNAs (shMYC1, 2) were used for each line, alongside a non-silencing (shNS) control (Supplementary Table 1). Across all 3 models, Dox-induced shMYC1 caused consistent MYC knockdown (KD) following 72 hours of Dox treatment (Figure 1B); MYC KD was further observed with shMYC2, but effects were more modest (Supplementary Figure 1A and B). As expected, shNS controls (+/−Dox) did not impact MYC expression (Figure 1B and C). Dox-induced shMYC1 resulted in slower proliferation observable in all 3 cell lines compared to untreated or shNS controls. In the D425med shNS + Dox condition, we observed an impact of Dox on proliferation at 120 hours; however, this was not comparable to the MYC KD-induced proliferation defect in D425med shMYC1 + Dox cells observable from 72 hours (Figure 1D and Supplementary Figure 1D). Similar trends were observed for shMYC2, consistent with the degree of MYC KD observed (Supplementary Figure 1C). These findings demonstrate ongoing dependency on MYC (ie, ‘MYC addiction’) for the growth of all 3 models and their relevance for the investigation of MYC-dependent biology. For subsequent experiments, we determined 72 hours of Dox treatment to be an effective timepoint where maximal MYC KD was achieved and there was the initiation of growth inhibition across the 3 MB_GRP3_ shMYC cell lines (Figure 1 and Supplementary Figure 1). Importantly, shNS controls provided a suitable background for distinguishing MYC-dependent effects from potential Dox-related treatment effects.

### MYC Drives Metabolic Alterations in MB_GRP3_ Cells

**Figure 2.**
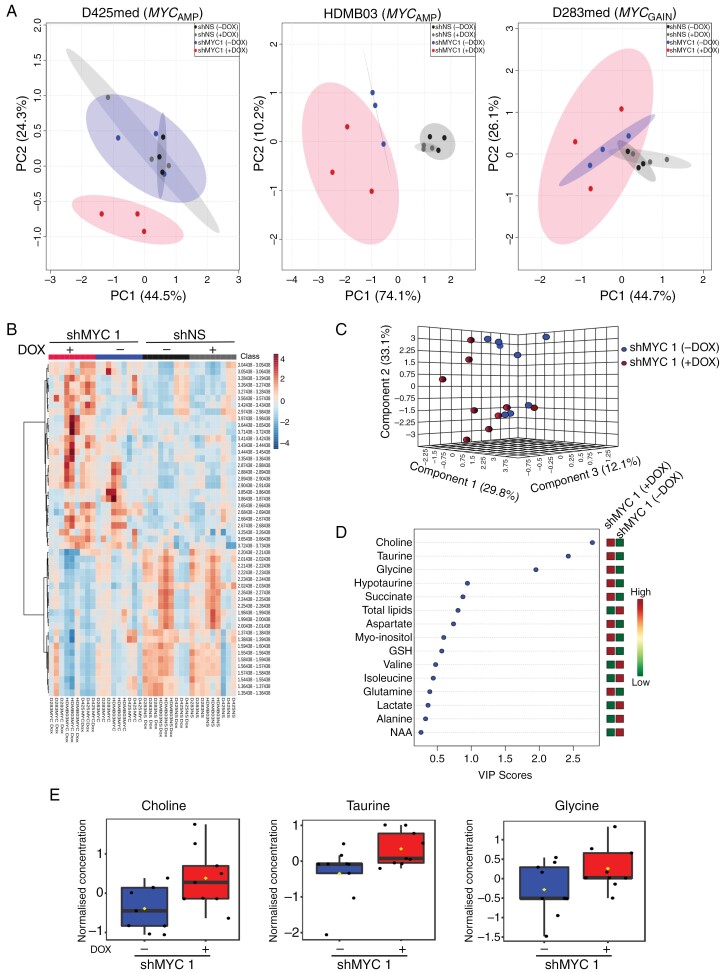
Perturbation of MYC expression alters the metabolite landscape of MB_GRP3_ cells. D425med, HDMB03, and D283med shNS and shMYC1 transduced cells were treated with 1 µg/mL Dox for 72 hours and subjected to metabolite profiling using ^1^H HRMAS. (A) Principal component analysis of spectral bins from HRMAS metabolite analysis, labeled by shRNA construct, and Dox treatment. (B) Hierarchically clustered heatmap analysis of relative spectral bin intensities of HRMAS spectra in MB_GRP3_ shNS and shMYC1 cells ± Dox treatment. (C) Partial least square discrimination analysis (PLS-DA) of identified metabolites from HRMAS spectra of the pooled D425med, HDMB03, and D283med shMYC1 ± Dox treated cells. (D) Variable importance (VIP) scores of the most significant metabolites contributing to the separating shMYC1 (−Dox) and shMYC1 (+Dox) groups as identified by PLS-DA. The red and green boxes to the right indicate whether a metabolite is increased (red) or decreased (green). (E) Normalized concentrations of the top 3 discriminant metabolites (VIP score ≥ 1.5) in the pooled MB_GRP3_ shMYC1 cells. Data indicates upper, median, and lower quartiles. Arbitrary units (AU). Data represents the means of 3 biological replicates.

We next sought to determine the impact of MYC on MB_GRP3_ metabolism. For this, we utilized ^1^H HRMAS to profile global metabolites and compared metabolite variations across our different models.^30^ In D425med cells, using spectral profiles, a single cluster containing shMYC1 MYC KD (ie, + Dox) cells, separated from MYC expressing shMYC1 (−Dox) and shNS (±Dox) clusters, indicating alterations in metabolites upon MYC KD. Similarly, HDMB03 and D283med shMYC1 (+Dox) cells separated from shMYC (−Dox) to varying degrees (Figure 2A). Heatmap analysis of spectral features further revealed robust changes in metabolite signatures following MYC KD with shMYC1 in all 3 MB_GRP3_ models. As anticipated, the spectral profiles of control shNS cells did not reveal clear or consistent changes (Figure 2B). Changes in the spectral profiles of shMYC2 cell lines were more modest, likely influenced by their less pronounced MYC KD (Supplementary Figure 2A and B). Together, these profiles reveal consistent MYC-dependent metabolic adaptations across independent MB_GRP3_ models.

From the observed HRMAS spectra, we quantified 21 specific metabolites and 5 lipid species (Supplementary Figure 3 and Table 2). We then combined individual shMYC1/2 according to ± Dox conditions and performed partial least-squares discriminant analysis (PLS-DA) to identify metabolites consistently altered by MYC modulation across all models (Figure 2C and Supplementary Figure 2C). Notably, increased levels of glycine and choline were strongly associated with MYC KD in both shMYC1 and shMYC2 cells. A similar increase in taurine was observed in shMYC1 and to a lesser extent in shMYC2 + Dox variants (Figure 2D and E, Supplementary Figure 2D and E and Supplementary Figure 3). Interestingly, these discriminant metabolites are all linked to one-carbon metabolism. The generation of glycine from serine is highly interlinked with fueling the folate and methione cycles and glutathione production downstream of the transsulfuration pathway.^31,32^ Although choline plays a role in membrane integrity, it can serve as a source for methyl groups for glycine production. Further downstream in the transsulfuration pathway, cysteine is generated from methionine and subsequently undergoes oxidization to produce taurine.^31^ In our own HRMAS data, whilst choline levels were increased in the shMYC KD cells, there were no consistent changes detected in other choline-containing metabolites (Supplementary Figure 3C-D). For taurine, we also assessed other related metabolites; both D425med shMYC1/2 and HDMB03 shMYC1 + Dox cells showed an increase in hypotaurine although this metabolite was not detectable in the D283med cell line (Supplementary Figure 3C-D). Since the metabolism of both choline and taurine can intersect with glycine metabolism, we focused on this particular metabolite for further investigation.

Glycine is a non-essential amino acid that can be synthesized de novo from glucose in the serine and glycine biosynthesis pathway (SGP) and is implicated in supporting cancer progression due to its major contribution of one-carbon units into nucleotide, protein, methylation, and redox homeostasis.^31–33^ We observed consistent alterations in glycine levels in both shMYC1 and shMYC2 conditions (the latter had considerably fewer metabolite alterations overall; Figure 2, Supplementary Figures 2 and 3). Given the reported direct transcriptional regulation of SGP genes by MYC,^34^ we hypothesized that the increased glycine levels within our MB_GRP3_ models following MYC KD, reflect distinct utilization/pathway dynamics and may be an important aspect of MYC-driven MB_GRP3_ metabolism.

### MYC Knockdown Impairs De Novo Serine and Glycine Synthesis

**Figure 3.**
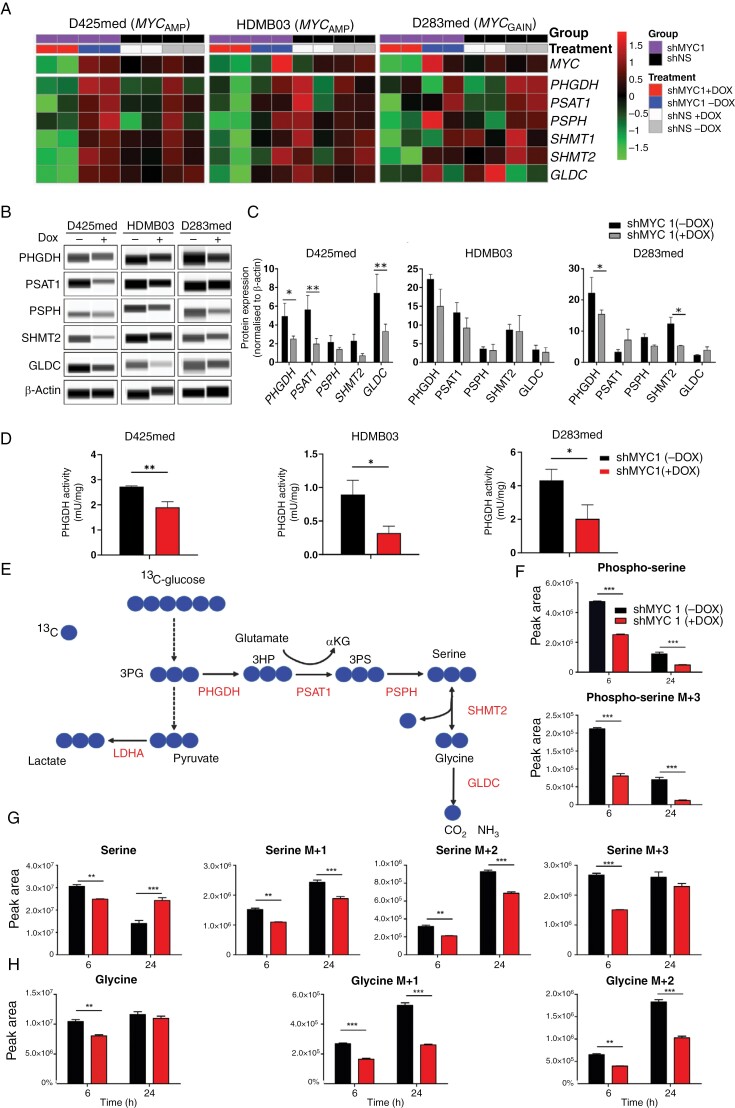
*MYC*-driven MB_GRP3_ cells display upregulation of de novo serine and glycine synthesis pathways. (A) Heatmaps visualizing log2 gene expression values for *MYC, PHGDH, PSAT1, PSPH, SHMT1, SHMT2,* and *GLDC* genes in MB_GRP3_ cell lines. Both shMYC1 and shNS groups are shown and their respective ± Dox conditions, in multiple independent replicates. PHGDH, phosphoglycerate dehydrogenase; PSAT1, phosphoserine aminotransferase 1; PSPH: phosphoserine phosphatase; SHMT1/2, serine hydroxymethyltransferase 1/2; GLDC, glycine decarboxylase. (B) Immunoblot analysis and (C) quantification of SGP pathway enzymes following MYC KD. β-actin was used as a loading control. Data represents mean ± SEM of 3 biological replicates. (D) Quantification of PHGDH activity in shMYC1 ± Dox. Mean ± SEM of 3 biological replicates. * *P* < .05, ** *P* < .01. (E). Schematic of isotopologues following ^13^C-glucose labeling in glycolysis and the de novo SGP. Peak areas of ^13^C isotopologues (F) phosphoserine (G) serine (H) glycine in D425med cells expressing shMYC1 following the addition of doxycycline and MYC knockdown. Metabolites are denoted with +1, +2, +3 to indicate the number of heavy isotopes. Data represents mean ± SD of 3 biological replicates- **P < .01, ***P < .001

MYC has previously been shown to transcriptionally activate SGP genes via binding to E-box elements leading to augmented expression.^34,35^ We therefore first assessed whether MYC KD led to the downregulation of SGP genes. Consistent with other reports, in our models downregulation of SGP enzymes corresponded to the severity of MYC KD which was most pronounced in *MYC*-amplified cell lines D425med and HDMB03 shMYC1, following Dox addition. In the *MYC* gained D283med, this effect was less striking although enzmes SHMT1/2 were noticeably downregulated upon MYC KD in the shMYC1 + Dox condition (Figure 3A). At the protein expression level, all MB_GRP3_ models displayed MYC-dependent alterations in SGP enzyme expression; most notably, reduced expression of PHGDH, the rate-limiting enzyme and committing step of SGP, was consistently observed following MYC KD (Figure 3B-C).^33^ These results indicate that MYC KD impacts both transcriptional and protein expression of SGP enzymes.

Next, we evaluated whether the activity of PHGDH itself, which determines the partitioning of glucose-derived intermediates towards SGP, was impeded following MYC KD. Here, we showed that indeed upon MYC KD, shMYC1 + Dox cells displayed lower PHGDH activity compared to MYC expressing shMYC—Dox cells (Figure 3D). Importantly in the shNS controls, Dox alone does not disrupt PHGDH activity (Supplementary 4A). Although we confirmed the MYC-dependent activity of PHGDH in our MB_GRP3_ cell lines, it was important to determine the contribution of de novo synthesis to intracellular levels of serine and glycine. We therefore employed uniformly labeled ^13^C-glucose tracing to assess glucose-derived serine and glycine synthesis; this method also enabled direct assessment of serine levels not readily detected by HRMAS. ^13^C-glucose partitioning into the de novo SGP gives rise to isotopologues reflecting the activity of the pathway (Figure 3E). Based on the detected ^13^C-glucose-derived isotopologues, we showed that across the entirety of the de novo *SGP,* levels of the fully labeled metabolites, phosphoserine M + 3, serine M + 3, and glycine M + 2 were markedly higher in MYC expressing cells compared to MYC KD and were most profound at the 6 hours timepoint. Through partially labeled isotopologues, we observed continued flux through the de novo SGP (Figure 3F-H). Additionally, higher levels of fully labeled lactate (M + 3) and pyruvate (M + 3) were observed in shMYC1 - Dox cells, indicative of greater turnover in the glycolysis pathway, consistent with observations from HRMAS (Supplementary Figures 4B and C and 3C-D). Together with HRMAS data, these data indicate faster glycolytic turnover and greater partitioning of glucose-derived intermediates in the SGP pathway in MYC-expressing cells compared to their MYC KD counterparts. It was interesting to note that levels of unlabeled serine significantly increased in MYC KD cells at 24 hours compared to 6 hours. Similarly, at 24 hours glycine levels were recovered and comparable to those in shMYC ± Dox conditions. This was interesting as it potentially pointed to exogenous contribution to serine and glycine pools following MYC KD.

To further expand on this, we next determined whether PHGDH was important for the proliferative ability of MYC-dependent MB_GRP3_ cells. We depleted PHGDH in all 3 MB_GRP3_ parental cell lines using CRISPR-mediated depletion (Supplementary Figure 4D). Depletion of PHGDH in D283med was lethal and no stable cell line could be generated in contrast to the non-targeting control (data not shown); it was therefore replaced with the *MYC*-amplified cell line, D458med. Following genetic depletion of PHGDH, we observed growth impairment across all 3 MYC-dependent cell lines suggesting that PHGDH is important in sustaining the proliferative capacity of MYC-driven MB_GRP3_ cells. (Supplementary Figure 4E-G). Furthermore, we undertook clonogenic assays, following the removal of serine, glycine, or both serine and glycine from the growth medium, to assess whether downregulation of PHGDH rendered MB_GRP3_ cells more dependent on exogenous sources of serine and glycine. Indeed, we found in corroboration with other studies, serine depletion was detrimental to PHGDH-depleted cells.^36^ Glycine removal alone did not impact clonogenicity as it can be synthesized from serine by SHMT. These data suggest upregulation of PHGDH by MYC-overexpressing MB_GRP3_ maintains sufficient intracellular levels of serine and glycine, uncoupling from requirements on exogenous availability to facilitate growth. Together, these data highlight a MYC-dependent increase in the flux of glucose-derived intermediates towards the de novo SGP, which coincides with increased expression and activity of PHGDH. Furthermore, PHGDH itself is important in maintaining the growth of MYC-dependent MB_GRP3_ cells.

### Upregulation of the De Novo SGP is a Targetable Vulnerability in *MYC*-Dependent MB_GRP3_ Cells

**Figure 4.**
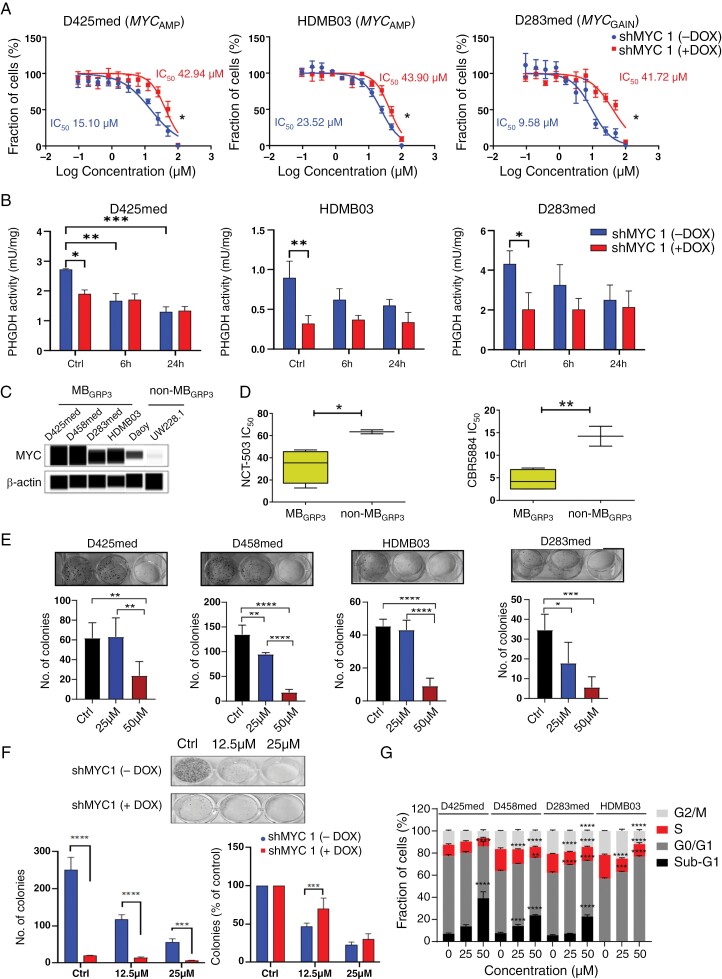
MB_GRP3_ cells are sensitive to pharmacological inhibition of the de novo SGP in a MYC-dependent fashion. (A) Dose–response curves following PHDGH inhibition mediated by NCT-503 treatment in shMYC1 isogenic cell lines ± Dox. Respective IC_50_ values are indicated. Confidence intervals D425med shMYC1 (− Dox) = 11.5–19.9 µM, D425med shMYC1 (+ Dox) = 32–57.4 µM. HDMB03 shMYC1 (− Dox) = 19.8–27.9 µM, HDMB03 shMYC1 (+ Dox) = 36.7–52.5 µM D283med shMYC1 (− Dox) = 6.7–13.6 µM, D283med shMYC1 (+ Dox) = 30.8–56.5 µM. Data represents the mean of 5 independent experiments ± SEM (B) Quantification of PHGDH activity following NCT-503 treatment in shMYC ± Dox conditions. Data expressed as mean ± SEM of 3 biological replicates. * *P* < .05, ** *P *< .01, *** *P* < .001. (C) Immunoblot analysis of MYC protein expression in *MYC*-amplified/gained MB_GRP3_ and non-amplified non-MB_GRP3_ cells. β-actin was used as a loading control. (D) Comparison of IC_50_ values of *MYC*-amplified MB_GRP3_ cells versus non-amplified non-MB_GRP3_ to PHGDH inhibitors, NCT-503 and CBR5884. Boxplot representation of IC_50_ values from 3 MB_GRP3_ and 2 non-MB_GRP3_ cell lines. IC_50_s derived from 3 independent experiments. The median with upper and lower quartiles are shown as appropriate (E). Representative images of clonogenic assays in D425med, D458med, D283med, and HDMB03 cell lines following 10-day treatment with 25 µM and 50 µM NCT-503. Quantifications represent means of 3 biological replicates ± SEM. Significance determined by one-way ANOVA. * *P* < .05, ** *P* < .01, *** *P* < .001, **** *P* < .0001. (F) Effect of NCT-503 treatment on colony formation on D425med MYC isogenic cells ± Dox treatment. Representative images of the clonogenic assay in D425med shMYC 1 cell ± Dox following 10-day treatment with 12.5 and 25 µM NCT-503 treatment. Colony numbers are shown normalized to their respective untreated controls. Data represents means of 3 biological replicates ± SEM. Significance determined by one-way ANOVA. *** *P* < .001, **** *P* < .0001. (G) Cell cycle analysis using propidium iodide staining following 72 hours of NCT-503 treatment. Data represents mean ± SEM from 3 biological replicates. Significance was tested using a two-way ANOVA for each cell line. * *P* < .05, ** *P* < .01. *** *P* < .001, **** *P* < .0001

We subsequently tested NCT-503,^37^ an allosteric inhibitor of PHGDH, in our 3 *MYC*-dependent MB_GRP3_ models. All MYC-expressing models were particularly sensitive to NCT-503 whereas MYC KD (shMYC1) rendered all cell lines less sensitive to SGP pathway perturbation (Figure 4A). We observed on-target PHGDH inhibition by NCT-503 in a time-dependent fashion across the 3 different shMYC1 − Dox, MYC expressing cells (Figure 4B). However, in the shMYC + Dox cells, we saw that relative PHGDH activity was not further perturbed by NCT-503 following MYC KD (Figure 4A and Supplementary Figure 5A). These findings suggest sensitivity to PHGDH inhibition by NCT-503 is dependent on the high basal MYC-dependent activity of PHGDH. We also tested NCT-503 and another inhibitor, CBR5884,^38^ in a panel of parental *MYC*-amplified/gained MB_GRP3_ cell lines alongside non-amplified/low MYC expressing non-MB_GRP3_ cells as comparators (Figure 4C). Similar to the isogenic cell lines, MYC-dependent cell lines were more sensitive compared to non-amplified non-MB_GRP3_ cells (Figure 4D and Supplementary Figure 5B and C). Subsequently, we determined the phenotypic consequences of PHGDH inhibition via NCT-503 treatment. Firstly, PHGDH inhibition impacted the colony formation of parental MB_GRP3_ MYC-overexpressing cell lines in a concentration-dependent manner, indicating long-term proliferative capacity was disrupted (Figure 4E). In the isogenic models, MYC KD severely impacted colony formation and there was a small additive effect of NCT-503 suggesting that the impact of PHGDH inhibition is more detrimental to high MYC-expressing cells NCT-503 (Figure 4F). Additionally, we observed higher proportions of cells in the sub-G1 and G1 phase of the cell cycle following NCT-503 treatment in MYC-driven MB_GRP3_ cell lines (Figure 4G). Taken together, our findings reveal MYC-dependent sensitivity to PHGDH inhibition in medulloblastoma cells resulting in reduced growth and proliferation.

### PHDGH Inhibition Slows Tumor Growth in In Vivo Preclinical Models and Represents a Candidate Targeted Therapy for *MYC*-Dependent MB_GRP3_

**Figure 5.**
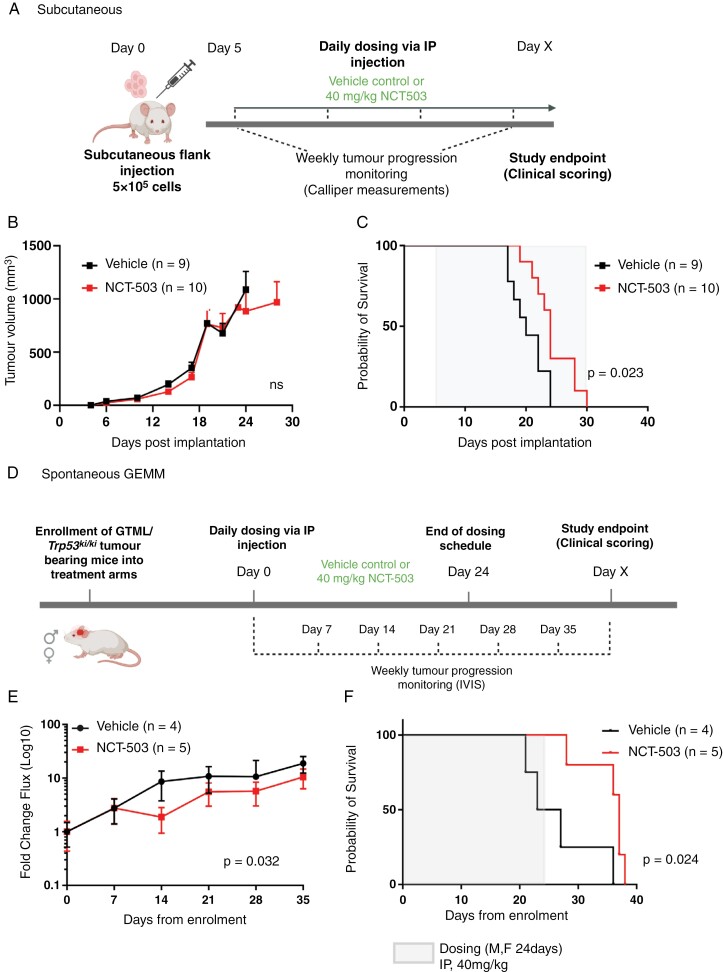
In vivo pro-survival effect of NCT-503 treatment. (A) Schematic diagram of in vivo NCT-503 treatment study in mice bearing subcutaneous xenografts (created using Biorender.com). (B) Tumor volumes of subcutaneous xenograft models established by flank injection of D425med in NSG mice. Mice with palpable tumors were injected with vehicle control or NCT-503 via IP injections. Data represents mean ± SEM. *n* = 8 per experimental group. (C) Kaplan–Meier survival curves of the vehicle or NCT-503 treated mice. (D) Schematic diagram of in vivo NCT-503 treatment study in a medulloblastoma GEMM model. Following spontaneous tumor establishment, mice were randomly assigned to teach treatment group (vehicle *n* = 4, NCT-503 *n* = 5) (created using Biorender.com). Mice received tumor monitoring by IVIS imaging on indicated days. Mice were euthanized after reaching humane endpoint. (E) Baseline corrected luminescence intensity from tumor-bearing mice. Data represents mean ± SEM. (F) Kaplan–Meier survival curves of vehicle or NCT-503 treated mice. * indicates *P* < .05

We next sought to determine the efficacy of PHGDH inhibition by NCT-503 on survival in vivo, using both subcutaneous xenograft and genetically engineered mouse models. NCT-503 is a (blood-brain barrier) BBB penetrant drug that accumulates in the brain following systemic administration.^37^ We established 40 mg/kg NCT-503 by intraperitoneal (IP) injection, as a tolerable dose in non-tumor-bearing mice indicating systemic PHGDH blockade was not detrimental to mouse health over the experimental period, consistent with other reports (Supplementary Figure 6A).^37,39^ Reflecting in vitro observations, NCT-503 significantly increased survival (i.e. time to reach humane endpoint) of mice bearing subcutaneous tumor xenografts of *MYC*-amplified MB_GRP3_ cells (D425med; *P* = .023; Figure 5A-C). We next used a genetically engineered model (GEMM) harboring spontaneous MYCN-driven MB_GRP3_ tumors (GTML; *Glt1-tTA/TRE-MYCN-Luc*), to better reflect innate tumor development and pharmacology.^40^ Furthermore, the GTML orthotopic model provided a model where there was an intact BBB. First, we assessed GTML-derived tumor neurospheres in vitro, which displayed sensitivity to NCT-503 (Supplementary Figure 6B). Additionally, NCT-503 treatment significantly inhibited PHGDH activity in GTML neurospheres (Supplementary Figure 6C and D). GTML mice with spontaneous medulloblastomas were given NCT-503 or vehicle IP injection daily for 24 days and followed to humane endpoint (Figure 5D). Findings were closely comparable to our xenograft model; NCT-503 moderately reduced the tumor burden of mice (*P* = .032) and mice had significantly longer survival (*P* = .024; Figure 5E and F). Thus, using 2 independent mouse models of MB_GRP3_, we show that PHGDH inhibition via NCT-503 slowed tumor progression and produced an overall survival benefit in tumor-bearing mice.

### Elevated De Novo SGP Genes Are a Clinically Relevant Feature of *MYC*-Amplified Group 3 Medulloblastoma

**Figure 6.**
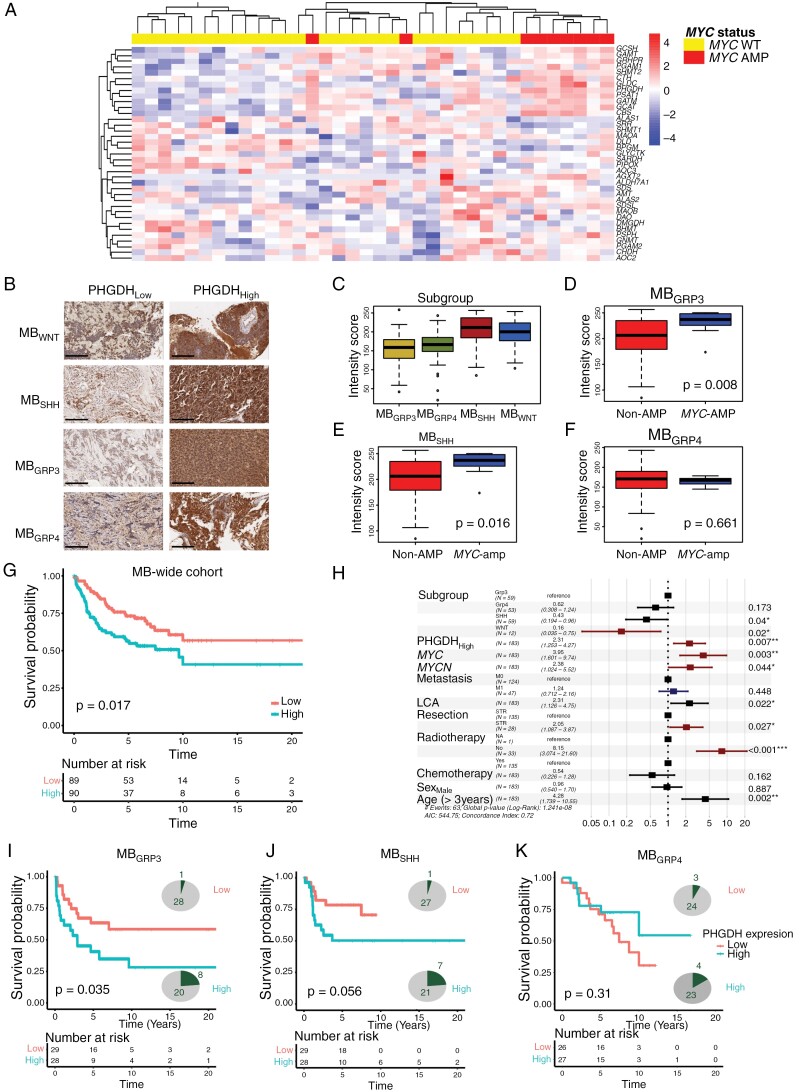
Upregulation of PHGDH is a clinically relevant feature in *MYC*-amplified medulloblastoma patient samples. (A) Hierarchical clustering and heatmap visualization of the SGP gene signature derived from the Cavalli et al., dataset. Expression values are z-score transformed; hierarchical clustering was performed using Ward distances. (B) Immunohistochemical staining of PHGDH in tissue microarrays containing the four molecular medulloblastoma subgroups samples, illustrating staining of PHGDH high and low samples (*n* = 183). Scare bar = 100 µM. (C) Quantification of PHGDH staining intensity from immunohistochemical analysis across MB_WNT_ (*n* = 12), MB_SHH_ (*n* = 58), MB_GRP3_ (*n* = 59), and MB_GRP4_ (*n* = 53) Boxplots display upper and lower quartiles and median of PHGDH intensity scores. (D-F) Subgroup-specific comparison of PHGDH intensity scores in *MYC*-amplified and non-amplified tumor samples in MB_SHH_ (*MYCN* amplified *n* = 8, non-amplified *n* = 50), MB_GRP3_ (*MYC* amplified *n* = 9, non-amplified *n* = 50) and MB_GRP4_ (*MYCN* amplified *n* = 7, non-amplified *n* = 49). Significance was determined using *t*-tests. (G) Kaplan–Meier survival curves of overall survival of medulloblastoma patients with PHGDH low and PHGDH high with a table depicting number of patients at risk at a given time period. Significance was tested using log-rank tests. (H) Forest plots of Cox proportional hazard models for the TMA, assessing PHGDH expression and other clinico-pathological parameters. The significance of hazard ratio estimates was evaluated by log-rank tests with * *P* < .05, *** *P* < .001. Red signifies features which are significant in univariate and multivariate analysis. Blue signifies features significant in univariate analysis only (I-K) Subgroup-specific Kaplan–Meier curves depicting association of PHGDH expression with overall survival. Indicated in the pie charts are the proportions of *MYC*-amplified (green) and non-amplified patients (gray) in the PHGDH_High_ and PHGDH_Low_ arms.

Our findings support a role for MYC-driven upregulation of SGP in MB_GRP3_. We therefore determined whether SGP dysregulation is a relevant feature of the primary human disease. We analyzed microarray data from the Cavalli et al., primary medulloblastoma cohort (*n* = 144) which defined MB_GRP3_ subtypes (Group 3α, 3β, 3γ) based on *MYC* status (balanced, loss, gain, and amplification) with the 3γ subtype containing most *MYC* gained/amplified samples.^2^ Here, we identified 17 genes associated with SGP (referred to as SGP gene signature) that were significantly altered across MB_GRP3_ (Supplementary Figure 7A). PHGDH was highly expressed in Group 3γ compared to Group 3α and 3β (Supplementary Figure 7B). We next interrogated whether this signature was specific to *MYC* amplification; for this, we used our independent MB_GRP3_ cohort (*n* = 36) with RNAseq data and *MYC* amplification status determined by DNA methylation array. We applied this gene signature for clustering-based analysis and found the SGP gene signature delineates *MYC*-amplified tumors from non-amplified tumors. We observed higher expression of SGP genes such as *PHGDH*, *SHMT2,* and *GLDC* in *MYC*-amplified tumors reinforcing our observations from the Cavalli et al. cohort^2^ (Figure 6A and Supplementary Figure 7A and B). Together, using 2 independent cohorts, we highlight that SGP enzymes are consistently differentially expressed in MYC-dependent MB_GRP3_ tumors compared to non-amplified tumors at the transcriptional level.

To specifically validate PHGDH as a therapeutically actionable target in MYC-driven MB, we assessed its protein expression in a large representative cohort of primary medulloblastomas (*n* = 183) and normal cerebellar controls (*n* = 9) (Supplementary Table 3). Overall, expression was higher in tumors compared to normal cerebellum (Supplementary Figure 7C-D). PHGDH expression was strongest in MB_WNT_ and MB_SHH_, with equivalent expression across MB_GRP3_ and MB_GRP4_ indicating cell of origin-linked variations (Figure 6B and C). Intra-group analysis (Figure 6D-F) revealed that *MYC* and *MYCN* amplification were associated with higher PHGDH expression in MB_GRP3_ and MB_SHH,_ respectively (Figure 6D and E). Notably, MYC and MYCN are both oncogenic drivers in these MB subgroups providing a crucial link between MYC pathogenicity in MB and PHGDH upregulation.^3,5^ In contrast, *MYCN*-amplification, not associated with increased risk in MB_GRP4,_^3,5,41^ was also not correlated with PHGDH expression in this group (Figure 6F).

Across our cohort, high PHGDH expression was associated with worse overall survival (OS; Figure 6G). Established risk features behaved as expected and high PHGDH expression was an independent risk factor in Cox-proportional hazard risk modeling (Figure 6H).^3^ Subgroup-specific interrogation showed high PHGDH expressers had worse outcomes in MB_GRP3_ and MB_SHH_, but not MB_GRP4_ (Figure 6I-H). Together our findings support the clinical relevance of PHGDH as a therapeutic target in medulloblastoma, with higher expression observed in *MYC-*amplified MB_GRP3_ defining a patient population that would most benefit from PHGDH perturbation. We also uncovered wider relevance in *MYCN-*amplified MB_SHH_ suggesting a mechanistic convergence of MYC-dependent pathways across poor-risk medulloblastoma groups.

## Discussion

Using steady-state HRMAS and SIRM metabolic profiling, we reveal the de novo SGP pathway as a novel targetable metabolic vulnerability driven by MYC in MB_GRP3_. In medulloblastoma and other MYC-driven cancers, direct MYC targeting remains challenging; however, MYC transcriptional co-dependencies provide potential alternative therapeutic strategies. We have demonstrated that MYC overexpression causes increased activity of the de novo SGP, mediated via PHGDH. MYC is a known transcriptional regulator of SGP enzymes via their E-box binding sites^35,39,42^; our findings thus extend these initial implications to highlight the de novo SGP as an important metabolic node which supports the proliferation of MYC-dependent MB_GRP3_.^39,43^

There remain limited options for personalized targeted therapies in medulloblastoma. Our data demonstrates MYC-induced metabolic vulnerabilities as a source of novel candidates for therapeutic interventions. Our initial NMR-based metabolic profiling detected a limited number of metabolites, thus utilizing broader metabolomics/lipidomic approaches may further illuminate MYC-dependent metabolic alterations not identified in this study. From our data, PHGDH has emerged as a promising metabolic node for targeting upregulated SGP in MYC-overexpressing MB_GRP3_ cells. Genetic depletion of PHGDH slows their growth and, alongside its pharmacological inhibition in multiple cell-based models, confirms a MYC-induced dependency with increased sensitivity to pathway perturbation. Furthermore, we found PHGDH expression is a representative biomarker and relevant therapeutic candidate associated with worse disease outcomes in *MYC*-amplified MB_GRP3_, providing the rationale for the pharmacological development of more potent inhibitors capable of BBB-penetrance. In addition, PHGDH was similarly overexpressed in high-risk *MYCN*-amplified MB_SHH_, but not in better prognosis *MYCN*-amplified MB_GRP4_, suggesting the pathogenicity of MYC in medulloblastoma subtypes correlates with upregulation of PHGDH and worse prognosis, and identifying a further medulloblastoma subgroup which may benefit from PHGDH targeting.

In vivo, administration of NCT-503 caused equivalent prolonged survival in MYC-dependent GEMM and xenograft MB_GRP3_ models. Together with the observed impact of NCT-503 on PHGDH activity in cell-based models, these findings are consistent with both tumor uptake and PHGDH inhibition in vivo. NCT-503 was effective in the micromolar range in our context. Beyond this proof of clinical concept, more potent inhibitors will be required for blockade of SGP activity within the brain microenvironment.^44^ Relatedly, preclinical studies in patient-derived xenograft models of MB_GRP3_, encompassing comprehensive pharmacokinetic and pharmacodynamic assessments, will be obligatory steps in concept development towards the clinic. Next-generation PHGDH inhibitors have shown greater potency and selectivity for PHGDH and, in combination with dietary depletion of serine and glycine, have yielded a more dramatic reduction in tumor growth.^43,45–48^

PHGDH inhibition represents a promising metabolic strategy for slowing tumor progression of *MYC*-amplified MB_GRP3_; however, combination therapies are more likely to achieve greater anti-tumor effects. Combining standard chemotherapy agents such as cytarabine and 5-flurouracil with PHGDH inhibition or dietary deprivation of serine/glycine has shown promising effects in leukemia and warrants further consideration in medulloblastoma.^49,50^ Furthermore, the crucial role that the SGP serves in biomass generation provides a rationale for combining pharmacological targeting of PHGDH with pathways that rely on this process, such as nucleotide synthesis and DNA damage repair.^50^

In summary, targeting metabolic vulnerabilities is emerging as a promising therapeutic strategy in medulloblastoma. Our study uncovers a dependency on the de novo SGP in the growth of MYC-driven MB_GRP3_ cells. Importantly, elevated PHGDH expression is a feature of primary *MYC*-amplified MB_GRP3_ tumors and correlates with adverse disease outcomes. Together, these findings pave the way for further exploration of direct targeting of SGP enzymes and, additionally, the impact of environmental serine and glycine deprivation, in blocking the growth of MYC-dependent MB_GRP3_.

## Supplementary material

Supplementary material is available online at *Neuro-Oncology* (https://academic.oup.com/neuro-oncology).

noae179_suppl_Supplementary_Material

## Data Availability

The data will be made available upon reasonable request to the corresponding author.
