# Supplementary material for: MYC-dependent upregulation of the de novo serine and glycine synthesis pathway is a targetable metabolic vulnerability in group 3 medulloblastoma
Source: Neuro Oncol. 2024 Oct 8;27(1):237–53. doi: 10.1093/neuonc/noae179 (PMC11726242; doi:10.1093/neuonc/noae179)
Supplement: noae179_suppl_Supplementary_Material [file noae179_suppl_supplementary_material.docx]

**Supplementary information**

**MYC-dependent upregulation of the *de novo* serine and glycine synthesis pathway is a targetable therapeutic vulnerability in Group 3 medulloblastoma**

Magretta Adiamah^1^, Bethany Poole^1^, Janet C. Lindsey^1^, Sarah Kohe^3^, Alaide Morcavallo^2^, Florence Burté^1^, Rebecca M. Hill^1^, Helen Blair^1^, Mankaran Singh^1^, Dean Thompson^1^, Shanel Swartz^1^, Stephen Crosier^1^, Tong Zhang^4^, Oliver D.K. Maddocks^4^, Andrew Peet^3^, Louis Chesler^2^, Ian Hickson^1^, Ross J. Maxwell^1^, Steven C. Clifford^1^

^1^Wolfson Childhood Cancer Research Centre, Newcastle University Centre for Cancer, Newcastle Upon Tyne, UK,

^2^Division of Clinical Studies, Institute of Cancer Research (ICR), London and Royal Marsden NHS Trust, Sutton, United Kingdom

^3^Institute of Cancer and Genomic Sciences, University of Birmingham, Birmingham, UK

^4^Institute of Cancer Sciences, University of Glasgow, Glasgow, UK

Correspondence:

Steve C. Clifford

Wolfson Childhood Cancer Research Centre

Newcastle University Centre for Cancer,

Herschel Building, Level 6, Brewery Lane,

Newcastle upon Tyne,

NE1 7RU, United Kingdom

Tel: +44 191 208 2239 (PA)

Email: steve.clifford@newcastle.ac.uk


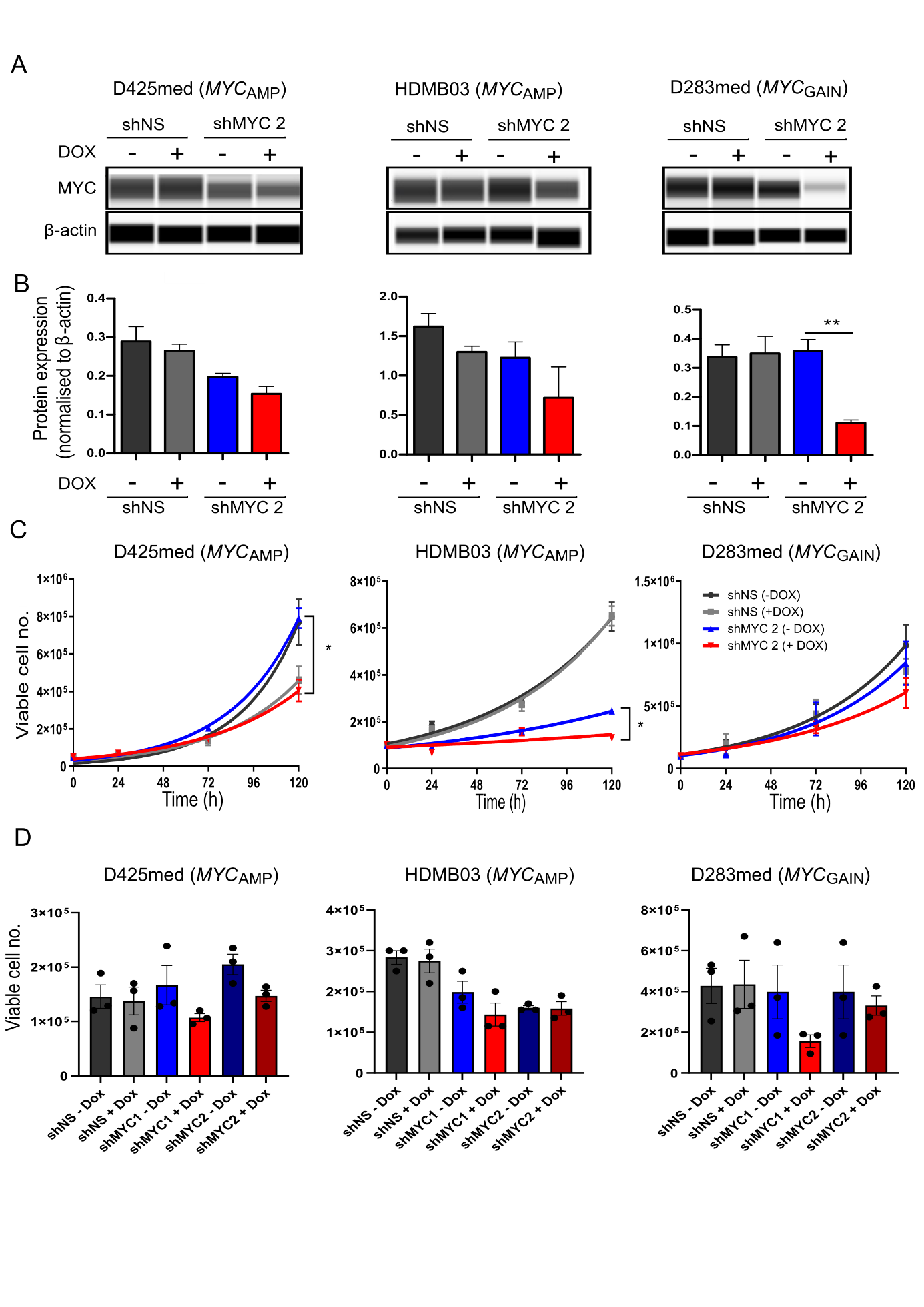


**Supplementary Figure 1.** Downregulation of MYC expression using the Tet-on system in MYC-driven MB_GRP3_ cells. A) Immunoblot analysis and B) quantification of MYC protein expression in D425med, HDMB03 and D283med MB_GRP3_ cells expressing shRNAs targeting non-silencing (shNS) and MYC (shMYC2) upon addition of 1µg/ml doxycycline (DOX) which induces MYC knockdown. β-actin was used as a loading control. C) Effect of MYC knockdown on cell proliferation was assessed using trypan blue exclusion viable cell counting over 120h. Growth curves depict D425med, HDMB03, D283med shNS and shMYC2 bearings cells ± Dox D) Viable cell number at 72h across MB_GRP3_ cell lines- Values are expressed as mean ± SEM of 3 biological replicates. *p<0.05, **p<0.01, ***p<0.001.

**
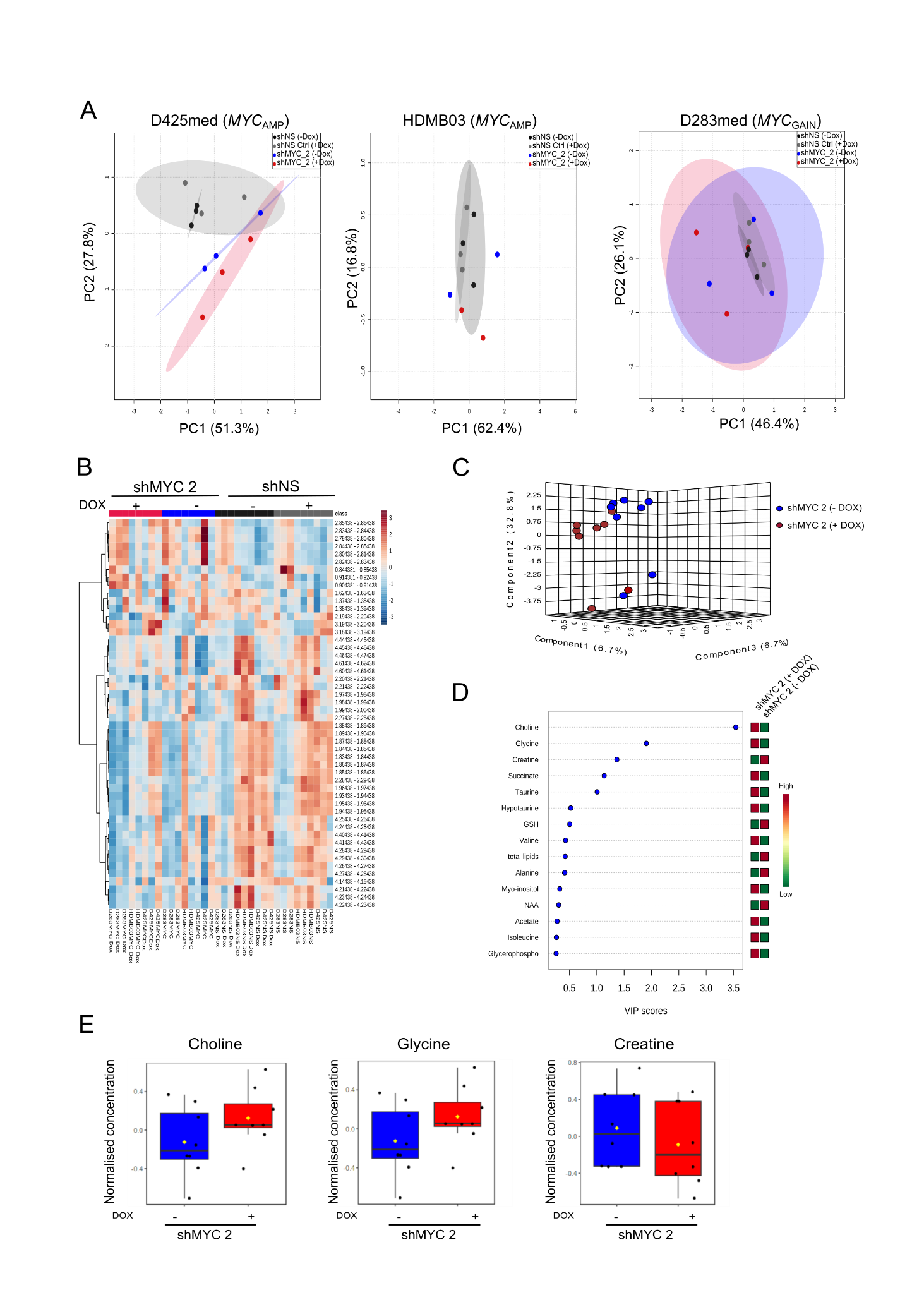
**

**Supplementary Figure 2.** MYC-dependent alterations in metabolites in MB_GRP3_ shMYC2 bearing cells. D425med, HDMB03 and D283med shNS and shMYC2 transduced cells were treated with 1µg/ml Dox for 72h and subjected to metabolite profiling using 1H HRMAS. A) Principal component analysis of spectral bins from HRMAS metabolite analysis, labelled by shRNA construct, and Dox treatment. B) Hierarchically clustered heatmap analysis of spectral regions in MB_GRP3_ shNS and shMYC2 cells ± Dox treatment. Colours represent relative spectral bin intensities. C) Partial least square discrimination analysis (PLS-DA) of identified metabolites from HRMAS spectra of the pooled D425med, HDMB03 and D283med shMYC2 ± Dox treated cells. D) Variable importance (VIP) scores of the most significant metabolites contributing to the separating shMYC2 (-Dox) and shMYC2 (+Dox) groups as identified by PLS-DA. The red and green boxes to the right indicate whether a metabolite is increased (red) or decreased (green). E) Normalised concentrations of top 3 discriminant metabolites in the pooled MBGRP3 shMYC1 cells (VIP scores ≥ 1.5). Data indicates upper, median and lower quartiles. Arbitrary units (AU). Data represents means of biological replicates D425med (n= 3), D283med (n= 3) and HDMB03 (n=2)

**
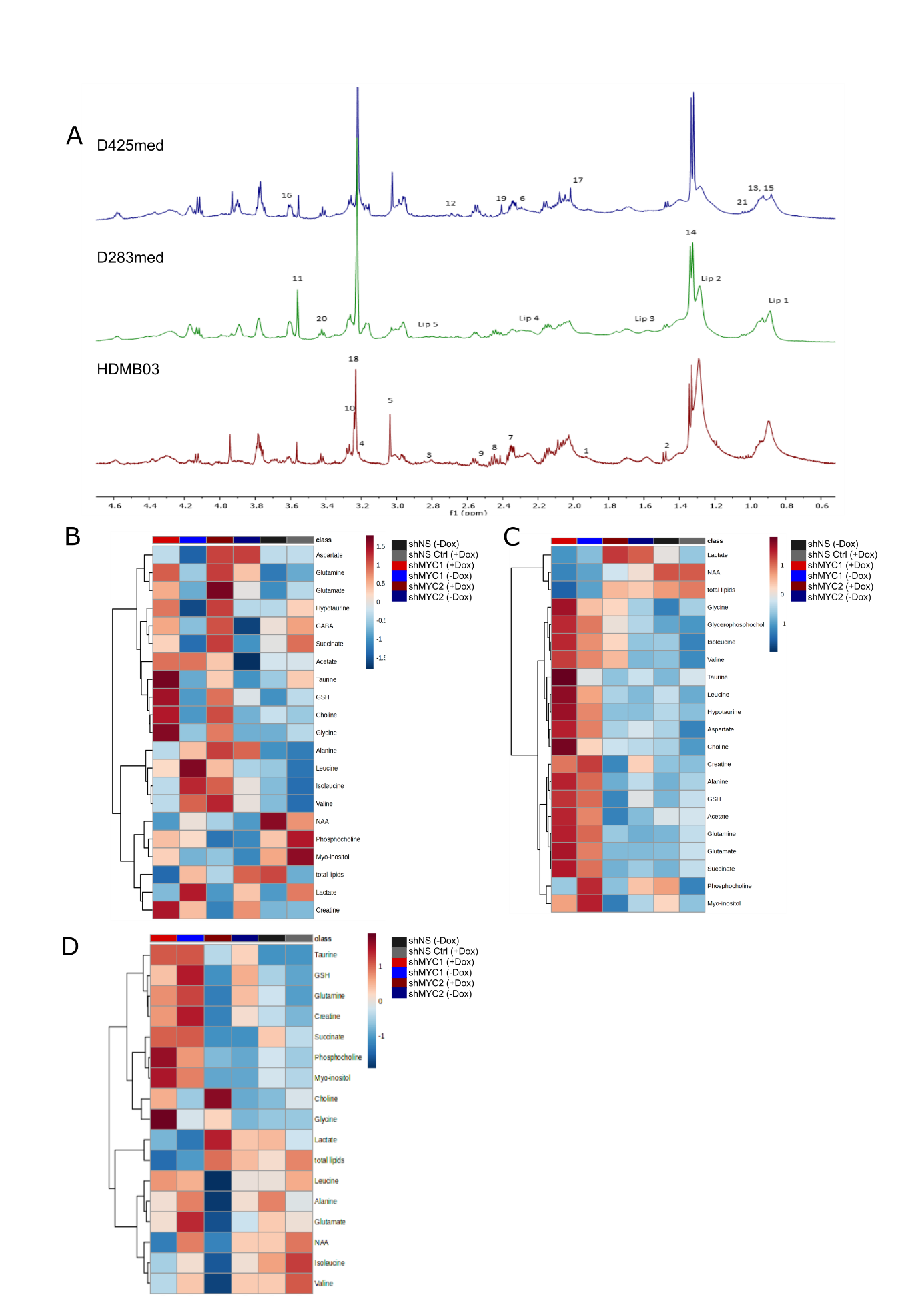
**

**Supplementary Figure 3**. Quantification of metabolites detected from HRMAS spectra. A) Representative HRMAS spectra for MBGRP3 cell lines indicating identified metabolites. Clustered heatmaps (Ward distances) showing the corresponding metabolites detected across B) D425med NS, shMYC1 and shMYC2 cells C) HDMB03 NS, shMYC1 and shMYC2 D) D283med NS, shMYC1 and shMYC2 following 1ug/ml doxycycline treatment over 72 hours. Data represents mean of 3 biological replicates for each condition except HDMB03 shMYC2 (n= 2).

**
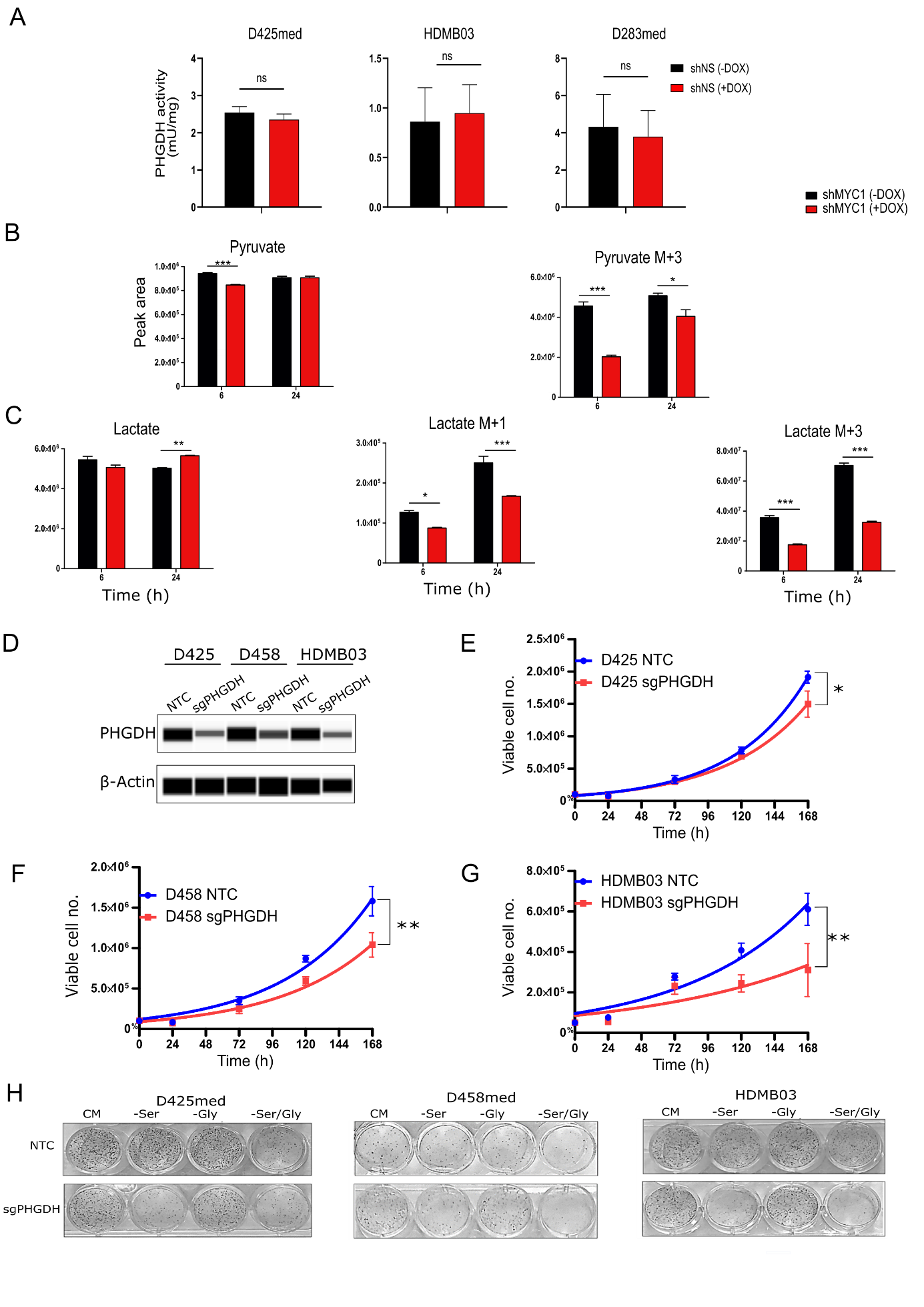
**

**Supplementary Figure 4.** *MYC*-driven dependence on the *de novo* serine and glycine synthesis pathway. A) Quantification of PHGDH activity in D425med, HDMB03, D283med shNS ± Dox conditions. Mean ± SEM of 3 biological replicates. B) Peak areas of ^13^C isotopologues C) Pyruvate D) Lactate in D425med shMYC1 cells following Dox treatment and MYC knockdown. Metabolites are denoted with +1, +2, +3 to indicate number of heavy isotopes. Data represents mean ± SD of 3 biological replicates- **p<0.01, ***p<0.001. E) Immunoblot analysis CRISPR-mediated knockdown of PHGDH in D425med, D458med and HDMB03. β-Actin was used as loading control. Growth curves of CRISPR-mediated knockdown of PHGDH in MB_GRP3_ cells lines in F) D425med G) D458med and H) HDMB03 using trypan blue dye exclusion. Values represent mean ± SEM of 3 biological replicates. *p<0.05, **p<0.01 I) Clonogenic assays of D425med, D458med, HDMB03 in complete medium (CM), serine (-Ser), glycine (-Gly) and combination (-Ser/-Gly) deprived conditions.


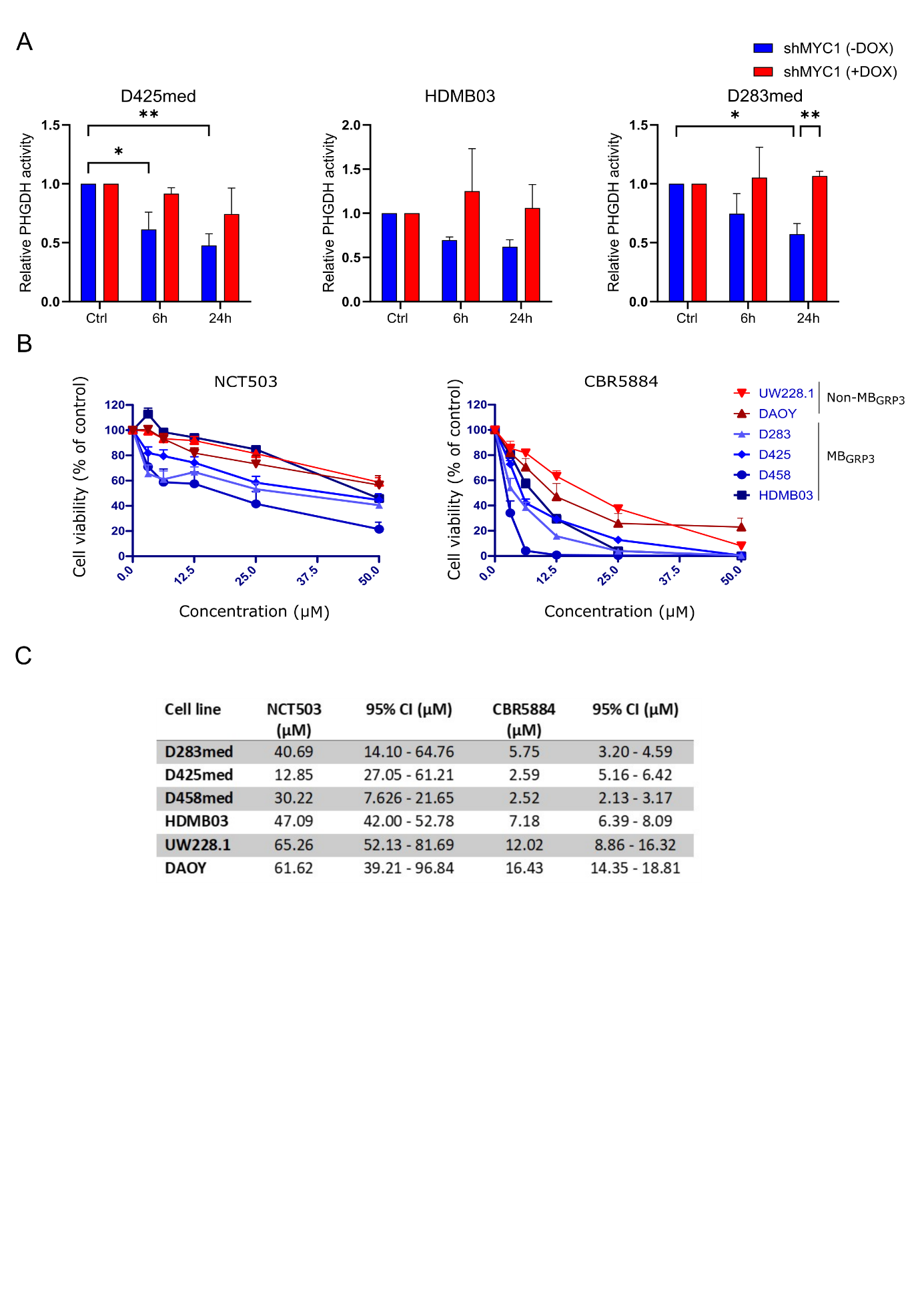


**Supplementary Figure 5.** Cytotoxicity of PHGDH inhibitors in a panel of medulloblastoma cell lines**.** A) Relative PHGDH activity in shMYC1 ± Dox following NCT-503 treatment. * p < 0.05, ** p < 0.01. B) Dose response curves of NCT-503 and CBR5884 treatment in parental MYC-dependent MB_GRP3_ (blue) and low MYC expressing non-MB_GRP3_ (red) cell lines. Line graphs show data from 6 independent experiments ± SEM C) IC_50_s of MYC-amplified/gain MB_GRP3_ cells versus non-amplified non-MB_GRP3_ cells to PHGDH inhibitors NCT-503 and CBR5884 with confidence intervals indicated.


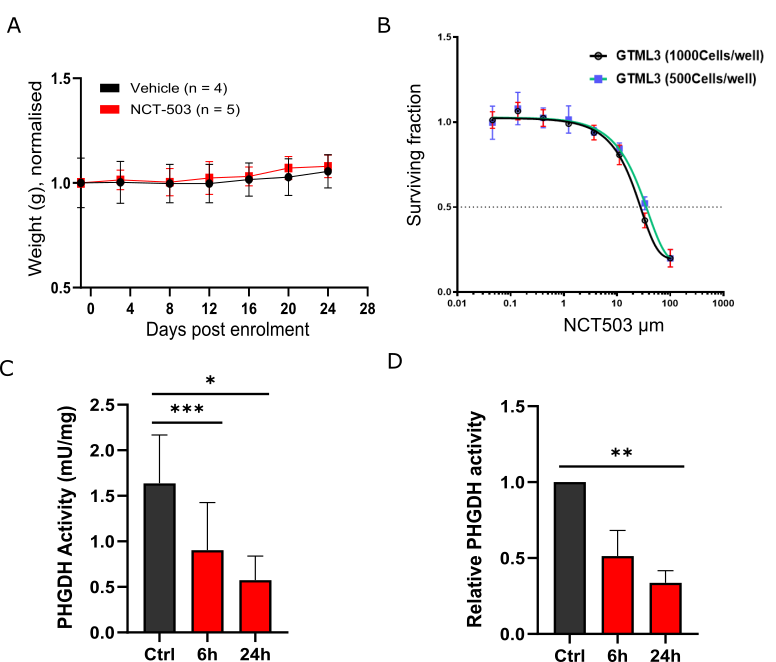


**Supplementary Figure 6.** Tolerability and in vitro efficacy of NCT-503 in GTML-derived neurospheres. A) Body weight measurements of non-tumour bearing NSG mice following systemic delivery of vehicle control (n= 3) and NCT-503 (n= 3) treated mice. B) *In vitro* dose response curves of NCT-503 sensitivity in GTLM^ki/ki^ derived neuropsheres. Cells were treated with NCT-503 for 120 hours and cell viability determined by celltiter glo. Data normalised as percentage of non-treated control. Data represents mean ± SEM of 3 biological replicates. C-D) Quantification of PHGDH activity following NCT-503 treatment in GTML neurospheres. Data expressed as mean ± SEM of 3 biological replicates. * p < 0.05, ** p < 0.01, *** p < 0.001.


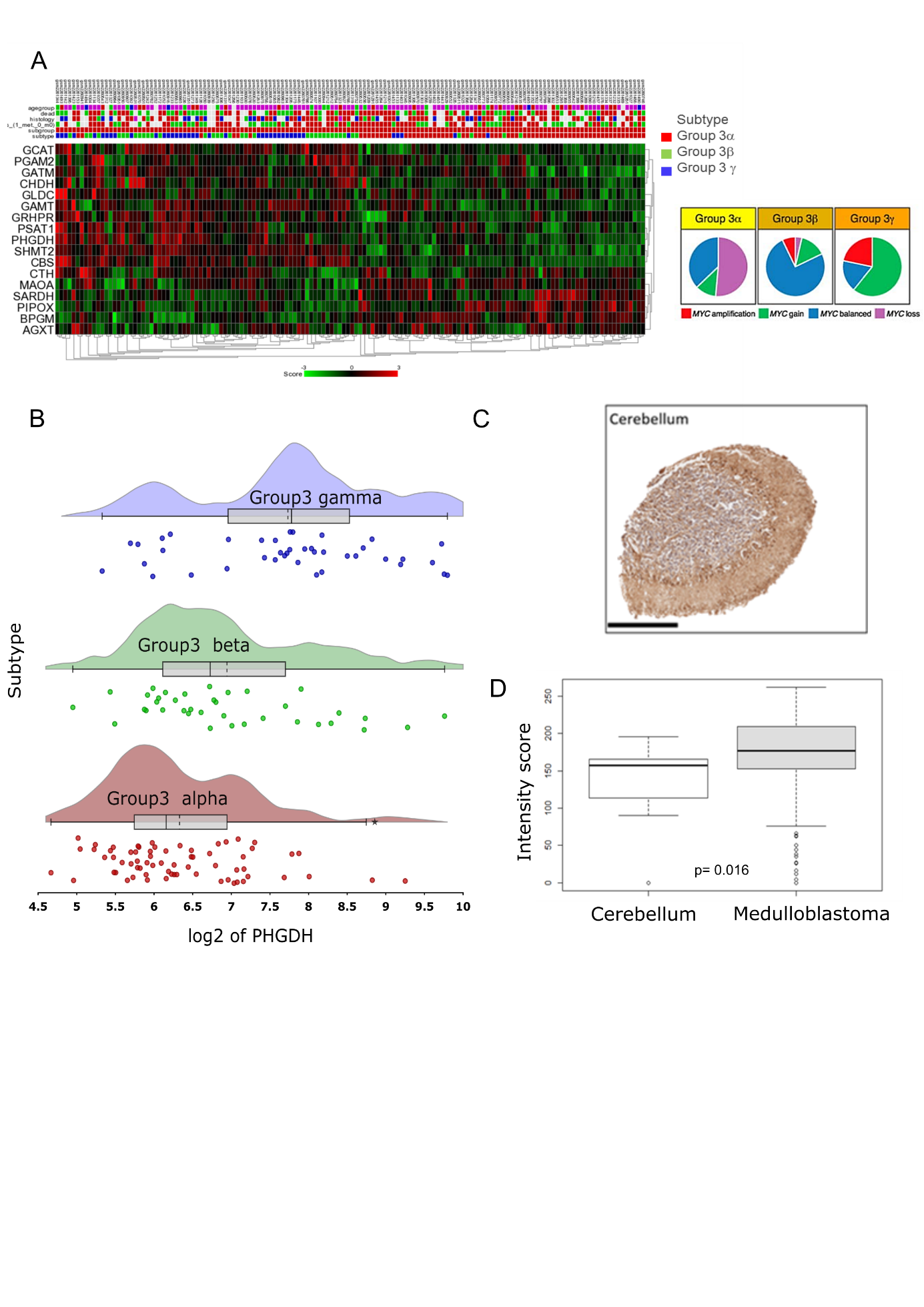


**Supplementary Figure 7.** Relevance of SGP in primary medulloblastoma. A) Gene expression profiling of the serine and glycine metabolism in the Cavalli *et al.* MB_GRP3_ dataset. Hierarchical clustering and heatmap visualisation of the 17 differential genes in the serine and glycine pathway in MB_GRP3_. Expression values are z-score transformed and hierarchical clustering performed using Euclidean distances. Colours represent Z-score values. MB_GRP3_ are further classified in the Cavalli cohort into 3 subtypes based on MYC features. B) PHGDH gene expression (log2) in Cavalli et al, defined Group 3 subtpes. Clouds depict the distrubution of samples and box plots depict median with lower and upper interquartiles. C) Representative PHGDH immunohistochemical staining of cerebellum control tissue. Scare bar = 300 µM. D) Comparison of PHGDH expression in cerebellum and medulloblastoma tumour samples. Boxplots displaying upper and lower quartiles and median of PHGDH intensity scores across cerebellum (n= 9) and medulloblastoma tumours including MB_GRP3_, MB_GRP4_, MB_SHH_ and MB_WNT_ tumours (n= 183). Scale bar represents 100 µM. Significance was determined using t test.

***Supplementary table 1.*** *List of shRNA sequences.*

| **Construct** | **Strand** | **Target sequence** |
| --- | --- | --- |
| Non-silencing | Forward | 5’CCGGCAACAAGATGAAGAGCACCAATCGAGTTGGTGCTCTTCATCTTGTTGTTTTT3’ |
|  | Reverse | 5’AATTAAAAACAACAAGATGAAGAGCACCAACTCGAGTTGGTGCTCTTCATCTTG TTG 3’ |
| shMYC1 | Forward | 5’CCGGGATGAGGAAGAAATCGATGCTCGAGCATCGATTTCTTCCTCATCTTTTT 3’ |
|  | Reverse | 5’AATTAAAAACAACAAGATGAAGAGCACCAACTCGAGTTGGTGCTCTTCATCTTGTTG 3’ |
| shMYC2 | Forward | 5’CCGGCCTGAGACAGATCAGCAACAACTCGAGTTGTTGCTGATCTGTCTCAGGTTTTT 3’ |
|  | Reverse | 5’AATTAAAAACCTGAGACAGATCAGCAACAACTCGAGTTGTTGCTGATCTGTCTCAGG 3’ |

|  | **Identified metabolite** | **Chemical shift (ppm)** |  | **Identified lipid group** | **Chemical shift (ppm)** |
| --- | --- | --- | --- | --- | --- |
| 1 | Acetate | 1.92 (s) | Lipid 1 | CH_3_ | 0.9 |
| 2 | Alanine | 1.48 (d) | Lipid 2 | CH2)N | 1.29 |
| 3 | Aspartate | 2.83 (dd) | Lipid 3 | CH2-CH2=O | 1.59 |
| 4 | Choline | 3.20 (s) | Lipid 4 | CH2CH2C=O | 2.26 |
| 5 | Creatine | 3.03 (s) | Lipid 5 | =CH- CH_2_ -CH= | 2.8 |
| 6 | GABA | 2.30 (t) |  |  |  |
| 7 | Glutamate | 2.34 (m) |  |  |  |
| 8 | Glutamine | 2.44 (m) |  |  |  |
| 9 | Glutathione | 2.55 (m) |  |  |  |
| 10 | Glycerophosphocholine | 3.23 (s) |  |  |  |
| 11 | Glycine | 3.56 (s) |  |  |  |
| 12 | Hypotaurine | 2.65 (t) |  |  |  |
| 13 | Isoleucine | 1.01 (d) |  |  |  |
| 14 | Lactate | 1.32 (d) |  |  |  |
| 15 | Leucine | 0.95 (t) |  |  |  |
| 16 | Myo-inositol | 3.62 (t) |  |  |  |
| 17 | N-acetylaspartate (NAA) | 2.02 (s) |  |  |  |
| 18 | Phosphocholine | 3.22 (s) |  |  |  |
| 19 | Succinate | 2.40 (s) |  |  |  |
| 20 | Taurine | 3.42 (t) |  |  |  |
| 21 | Valine | 1.07 (d) |  |  |  |

***Supplementary table 2.*** *List of identified metabolies and lipids from HRMAS spectra.*

***Supplementary table 3.*** *Medulloblastoma tissue microarray cohort demographics compared to Schwalbe et al. cohort.*

|  |  | **TMA cohort** | **Schwalbe *et al.* 2017** |
| --- | --- | --- | --- |
| Cohort size |  | n= 183 | n= 428 |
| Age at diagnosis (median) |  | 5.36 | 6.34 |
| Sex | M | 119 (66%) | 278 (65%) |
|  | F | 60 (34%) | 150 (35%) |
| Resection | GTR | 134 (83%) | 285 (74%) |
|  | STR | 27 (17%) | 98 (26%) |
| Histology | CLA | 104 (62%) | 276 (70%) |
|  | DN/MBEN | 41 (24%) | 58 (15%) |
|  | LCA | 21 (13%) | 60 (15%) |
|  | MBNOS | 2 (1%) | 34 |
| MYC | Amplified | 12 /7%) | 22 (5%) |
|  | Non-amplified | 171 (93%) | 404 (95%) |
| MYCN | Amplified | 15 (8%) | 29 (7%) |
|  | Non-amplified | 168 (92%) | 397 (93%) |
| Metastatic stage | M+ | 47 (28%) | 104 (27%) |
|  | M0 | 123 (72%) | 285 (73%) |
| Subgroup | WNT | 12 (7%) | 33 (8%) |
|  | SHH | 58 (32%) | 109 (26%) |
|  | GRP3 | 59 (32%) | 130 (31%) |
|  | GRP4 | 53 (29%) | 153 (36%) |

**Supplementary methods**

**Immunoblotting**

Target specific antibodies were: MYC (Cell signalling #18583), PHGDH (Proteintech, UK #14719-1-AP), PSAT1 (Proteintech, UK #10501-1-AP), PSPH (Proteintech, UK #14513-1-AP), SHMT2 (Proteintech, UK #11099-1-AP), GLDC (Proteintech, UK #24827-1-AP), pS6 (Cell signalling, UK #35708), p4EBP1 (Cell Signalling Technology, UK # #39788), β-actin (Abcam, UK ab8227).

**HRMAS acquisition**

^1^H spectra was acquired in a 500 MHz Bruker AVANCE spectrometer^30^. The rotor was inserted into 4mm 3 channel 1H HRMAS z-PFG ban probe in a 500 MHz Bruker AVANCE spectrometer (Bruker, Coventry, UK). ^1^H NMR spectra were acquired at 54.7° magic angle at 4°C at rotor spin speed on 4 Khz. A standard NOESY sequence with water pre-saturation was acquired with a relaxation delay of 2 s and repetition time of 4 s. Each free induction decay (FID) was Fourier transformed, phased and baseline corrected. Metabolites were identified and quantified using Mestresnova v.9.01 (Mestrelab Research, Santiago, Spain). Metabolites were assigned based on the literature^30, 33^ and the human metabolome database^34^. Metaboanalyst version 4 was used for multivariate and clustering analysis^35^. Metabolite assignments are outlined in supplementary table 1. Metabolites were normalised normalised to sum of metabolites. *Metabolic profile analysis.* The scores from the first two Principal Components (PC1 and PC2) were used for clustering for individual shMYC1/2 cell lines following doxycycline treatment. To identify MYC-specific alterations, shMYC1/2 were combined across D425med, D283med and HDMB03 cell lines in their respective doxycycline conditions. Partial least-squares discriminant analysis (PLS-DA) was to cluster shMYC1/2 (± Dox) cells using 3 PLS-DA components. The PLS-DA feature, variable importance projection (VIP) scores was used to identify MYC-specific metabolite alterations.

**PHGDH activity assay**

MB cells and GTML neurospheres were treated with 1 x IC_50_ of NCT-503 (Sigma; SML1659) or vehicle control for 6 or 24 hours. Cell pellets were collected, washed twice with ice-cold PBS and flash frozen in dry ice.

**^13^C-glucose tracing**

Cells were pre-treated with ± 1 µg/mL of dox for 48 and subsequently plated in 6-well plates and further treated for a 24h ± 1 µg/mL doxycycline. Growth medium was removed from each well, washed with 1 x PBS and replaced with 10 mM D-glucose-^13^C (Sigma Aldrich, UK) or unlabelled glucose containing medium for 6 or 24h. ^13^C labelled and unlabelled samples were washed once with ice cold PBS. For metabolite extraction, a mixture containing methanol: acetonitrile: water (50:30:20 v/v) was used and the volume used was adjusted to obtain 2 x 10^6^ cells/ml. Cells were scraped into cold extraction mixture on ice, homogenised and centrifuged 15000 g for 15 min at 4°C to pellet the cell debris. The supernatant was stored - 80°C.

Analytes were separated using liquid chromatography with SeQuant ZIC pHILIC column (2.1 x 150 mM, 5 µM) coupled to a SeQuant ZIC pHILIC guard column (2.1 x 20 mM, 5 µM) and detected using high resolution MS with Orbitrap Exactive (Thermo Scientific) in line with a Aceela autosampler and Aceela 600 pump (Thermo Scientific). The Exactive operated using polarity switching mode with positive voltage at 4.5 kV and negative of 3.5 kV. Flow rate was 100 µl min-1, buffers consisted of acetonitrile (CAN) for A and 20 mM (NH4) CO3 in addition with NH4OH in dH20 for B. Gradient ran from 80% (v/v) to 405 CAN in 20 min followed by was at 20% (v/v) CAN and re-equilibration at 80% (v/v) CAN. LCquan (Thermo Scientific) was used for metabolites were quantification. Positive identification of metabolites was based on exact mass within 5 ppm, which was further validated by concordance with standard retention times and finally plotted as peak area for each metabolite.

**RNA sequencing**

Sequencing reads were aligned to the hg19 reference genome using RNA-Star and gene counts (Gencode v25 annotation) calculated using ht-seq. For each cell line, read counts for all samples were normalized and log2 transformed using DESeq2. Transformed counts for *MYC, PHGDH, PSAT1, PSPH, SHMT1, SHMT2* and *GLDC* were then visualised via heatmap using the pheatmap package (R/Bioconductor).

**Generation of PHGDH knockout cell lines**

Group 3 medulloblastoma cell lines (D425med, D458med, D283med, and HDMB03) were transduced with pLentiCRISPR non-targeting control (a gift from Paul Sinclair, Newcastle University UK) or pLentiCRISPR-PHGDH (a gift from David Sabatini, Addgene plasmid #83913) by lentiviral infection and underwent selection under 1µg/ml puromycin for 7 days. Bulk cell populations were assessed for target depletion using immunoblotting.
